# Controlling Pericellular Oxygen Tension in Cell Culture Reveals Distinct Breast Cancer Responses to Low Oxygen Tensions

**DOI:** 10.1002/advs.202402557

**Published:** 2024-06-14

**Authors:** Zachary J. Rogers, Thibault Colombani, Saad Khan, Khushbu Bhatt, Alexandra Nukovic, Guanyu Zhou, Benjamin M. Woolston, Cormac T. Taylor, Daniele M. Gilkes, Nikolai Slavov, Sidi A. Bencherif

**Affiliations:** ^1^ Department of Chemical Engineering Northeastern University Boston MA 02115 USA; ^2^ Department of Bioengineering Northeastern University Boston MA 02115 USA; ^3^ Department of Pharmaceutical Sciences Northeastern University Boston MA 02115 USA; ^4^ Conway Institute of Biomolecular and Biomedical Research and School of Medicine University College Dublin Belfield Dublin D04 V1W8 Ireland; ^5^ Department of Oncology The Sidney Kimmel Comprehensive Cancer Center The Johns Hopkins University School of Medicine Baltimore MD 21321 USA; ^6^ Cellular and Molecular Medicine Program The Johns Hopkins University School of Medicine Baltimore MD 21321 USA; ^7^ Department of Chemical and Biomolecular Engineering The Johns Hopkins University Baltimore MD 21218 USA; ^8^ Johns Hopkins Institute for NanoBioTechnology The Johns Hopkins University Baltimore MD 21218 USA; ^9^ Departments of Bioengineering Biology Chemistry and Chemical Biology Single Cell Center and Barnett Institute Northeastern University Boston MA 02115 USA; ^10^ Parallel Squared Technology Institute Watertown MA 02472 USA; ^11^ Harvard John A. Paulson School of Engineering and Applied Sciences Harvard University Cambridge MA 02138 USA; ^12^ Biomechanics and Bioengineering (BMBI) UTC CNRS UMR 7338 University of Technology of Compiègne Sorbonne University Compiègne 60203 France

**Keywords:** anoxia, breast cancer, cancer metabolism, cell culture, hypoxia, hypoxia‐inducible factors, oxygen, physioxia

## Abstract

In oxygen (O_2_)‐controlled cell culture, an indispensable tool in biological research, it is presumed that the incubator setpoint equals the O_2_ tension experienced by cells (i.e., pericellular O_2_). However, it is discovered that physioxic (5% O_2_) and hypoxic (1% O_2_) setpoints regularly induce anoxic (0% O_2_) pericellular tensions in both adherent and suspension cell cultures. Electron transport chain inhibition ablates this effect, indicating that cellular O_2_ consumption is the driving factor. RNA‐seq analysis revealed that primary human hepatocytes cultured in physioxia experience ischemia‐reperfusion injury due to cellular O_2_ consumption. A reaction‐diffusion model is developed to predict pericellular O_2_ tension a priori, demonstrating that the effect of cellular O_2_ consumption has the greatest impact in smaller volume culture vessels. By controlling pericellular O_2_ tension in cell culture, it is found that hypoxia vs. anoxia induce distinct breast cancer transcriptomic and translational responses, including modulation of the hypoxia‐inducible factor (HIF) pathway and metabolic reprogramming. Collectively, these findings indicate that breast cancer cells respond non‐monotonically to low O_2_, suggesting that anoxic cell culture is not suitable for modeling hypoxia. Furthermore, it is shown that controlling atmospheric O_2_ tension in cell culture incubators is insufficient to regulate O_2_ in cell culture, thus introducing the concept of pericellular O_2_‐controlled cell culture.

## Graphical abstract

This study demonstrates that in O_2_‐controlled cell culture systems, cellular O_2_ consumption consistently leads to pericellular anoxia under physioxic and hypoxic conditions. Various factors, including cell culture medium volume, vessel type, cell density, and cell type significantly influence the resultant pericellular O_2_ levels. Consequently, a pericellular O_2_‐controlled cell culture approach has been developed to investigate breast cancer responses to low O_2_.

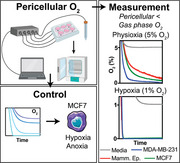

## 1 Introduction

A cornerstone of biological research, cell culture aims to grow cells in conditions that simulate their native environment as closely as possible. Cell culture models serve as a tool for testing biological hypotheses before validating in vivo. Healthy and diseased tissues are isolated from patients and studied in vitro. In fact, cell culture techniques are used throughout the process of drug development to make “go/no‐go” decisions^[^ ^1^, ^2^ ^]^ and to manufacture adoptive cell therapies and regenerative medicines.^[^ ^3^, ^4^ ^]^ Recent advances in this practice include scaffolds that mimic the extracellular matrix,^[^ ^5^, ^6^, ^7^ ^]^ self‐assembly of pluripotent stem cells to form brain organoids,^[^ ^8^ ^]^ patient‐derived organoids that capture tumor heterogeneity in patients and predict therapeutic responses, etc.^[^ ^9^, ^10^ ^]^ Yet, one key discrepancy between in vitro and in vivo remains: the “normoxic” (i.e., room air) O_2_ tension in cell culture (141 mmHg) is dramatically higher than the O_2_ tension of human tissues (3–100 mmHg) (1% O_2_ = 7.7 mmHg O_2_ at sea level).^[^ ^11^, ^12^, ^13^, ^14^, ^15^, ^16^, ^17^ ^]^

Supraphysiological O_2_ concentrations (hyperoxia) lead to excessive reactive oxygen species (ROS) production, resulting in cellular damage and dysregulated signaling.^[^ ^18^, ^19^ ^]^ It is therefore not surprising that cells grown in physioxia experience less oxidative stress.^[^ ^20^, ^21^ ^]^ Furthermore, hyperoxia degrades proteins containing a specific iron‐sulfur cluster, disrupting diphthamide synthesis, purine metabolism, nucleotide excision repair, and electron transport chain (ETC) function.^[^ ^22^ ^]^ Many O_2_‐dependent enzymes require iron and copper metal cofactors, which are susceptible to oxidation in hyperoxia.^[^ ^12^, ^23^, ^24^ ^]^ Studies culturing cells in normoxia versus physioxia have found aberrant T‐cell activation,^[^ ^25^ ^]^ fibroblast senescence^[^ ^26^ ^]^ and mutation frequency,^[^ ^27^ ^]^ chondrocyte differentiation,^[^ ^28^ ^]^ etc. in normoxia. However, the full impact of culturing cells in normoxia will remain unknown until physioxia becomes common practice.

To address these concerns, tools to perform physiological cell culture have been developed and are commercially available. These products, including portable chambers, tri‐gas incubators, and hypoxic workstations, consist of chambers that control O_2_ in the atmosphere of cultured cells by adding compressed nitrogen. However, widespread adoption has been hampered by cost, laboratory space requirements, technical challenges, and rapid reoxygenation of cultures.^[^ ^17^, ^29^ ^]^ Reoxygenation, which occurs when cell cultures are taken out of portable chambers or tri‐gas incubators and exposed to normoxia, makes it difficult to recapitulate physiological O_2_ tensions.^[^ ^23^, ^30^ ^]^ Hypoxic workstations address this issue effectively. One major application for these products is hypoxic cell culture models, conducted at 0.5–1% O_2_.^[^ ^31^, ^32^, ^33^, ^34^, ^35^ ^]^ Hypoxia occurs in both physiological (e.g., placenta, renal medulla, intestinal mucosa, germinal centers, bone marrow) and pathophysiological (e.g., infection, inflammation, solid tumors, ischemia) contexts; and is therefore, an active area of research.^[^ ^36^, ^37^ ^]^ Hypoxic cell culture was instrumental in the discovery of the prolyl hydroxylase (PHD)/hypoxia‐inducible factor (HIF) axis, the mechanism by which cells sense and respond to low O_2_.^[^ ^38^, ^39^ ^]^ O_2_‐controlled cell culture is also performed to mimic physioxia, typically at 5% O_2_.^[^ ^20^, ^21^, ^25^, ^28^ ^]^

In O_2_‐controlled cell culture, it is generally presumed that the atmospheric O_2_ tension within incubators is equal to the pericellular O_2_ tension, the O_2_ concentration that cells experience. The pericellular O_2_ tension is dependent on several rates: the O_2_ diffusion within the cell culture media, O_2_ transfer at the gas–media interface, and the cellular O_2_ consumption. Gas–media O_2_ transfer is the limiting rate.^[^ ^40^, ^41^ ^]^ Culture vessel geometry, medium volume, and surface area also influence diffusion times. These parameters vary greatly based on user preference and experimental design, yet they are not reported. Experimental studies measuring pericellular O_2_ tension indicate that confluent normoxic cultures can induce hypoxia.^[^ ^42^ ^]^ However, the impact of O_2_ consumption rates in lower O_2_ tensions is unclear, since consumption decreases as O_2_ becomes limiting.^[^ ^43^ ^]^ We set out to assess how key cell culture parameters (i.e., cell type, cell density, medium volume, and culture vessel geometry) influence the relationship between atmospheric and pericellular O_2_ tensions in O_2_‐controlled cell culture models. The theoretical nature of this relationship has been discussed in previous studies,^[^ ^17^, ^29^, ^44^, ^45^ ^]^ but experimental data supporting it are lacking. After discovering that pericellular O_2_ tension is vastly different from atmospheric O_2_ tension, we explored how controlling pericellular O_2_ tension could be used as a novel tool to study breast cancer cell responses in low O_2_.

## 2 Results

### 2.1 1% O_2_ Media Conditioning can Take over 5 Days and is Reoxygenated within Minutes

**Figure 1:**
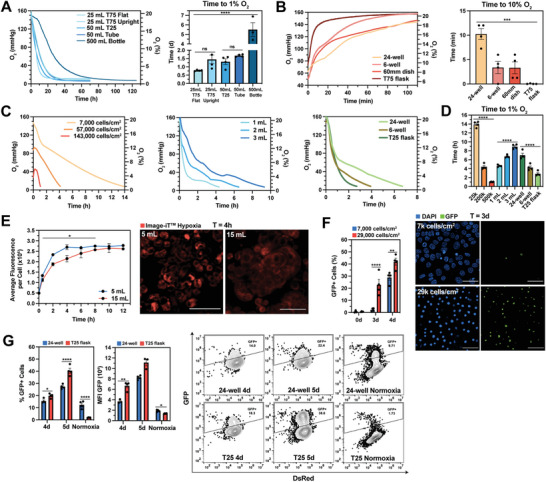
Cell culture parameters influence HIF stabilization kinetics in hypoxic (1% O_2_) culture. A) O_2_ kinetics (left) and time to 1% O_2_ (right) of normoxic media (EMEM + 10% FBS + 1% P/S) placed in a 1% O_2_ incubator. B) O_2_ kinetics (left) and time to 10% O_2_ (right) of 1% O_2_ media placed into different culture vessels under normoxia. C) O_2_ kinetics of MCF7 cultures with varying cell densities (left), medium volumes (middle), and culture vessel type (right) placed in a 1% O_2_ incubator. D) Time to 1% O_2_ from (C). E) Average fluorescence (Image‐iT^TM^ Hypoxia) per cell (left) and representative confocal images at 4 h (right) for MCF7 cultures (60mm dish) with 5 or 15 mL of media placed in a 1% O_2_ chamber. Red = Image‐iT^TM^ Hypoxia as an indicator of cellular hypoxia. F) Percentage of GFP+ cells (left) and representative confocal images at 3 days (right) from MCF7 HIF reporter cells cultured at 7,000 or 29,000 cells cm^−2^ for 4 days in a 1% O_2_ incubator. Blue = nuclei stained with DAPI, Green = GFP+ (HIF+) cells. G) Percentage of GFP+ cells (left), GFP mean fluorescence intensity (MFI) (middle), and representative contour plots with outliers (right) for MDA‐MB‐231 HIF reporter cells cultured in a 24‐well plate or T25 flask for up to 5 days in a 1% O_2_ incubator. Data were analyzed with ANOVA and Geisser‐Greenhouse (E) or Bonferroni (F‐G) corrections. N = 3–4 biological replicates per condition.

For O_2_‐controlled cell culture experiments, media is typically conditioned to the desired O_2_ tension and added to the cells at the start of the experiment. This procedure ensures that cells experience the desired O_2_ tension immediately. We investigated how long it would take to condition 25–500 mL of media for hypoxic (1% O_2_) experiments, since conditioning times are not reported.^[^ ^31^, ^33^, ^46^ ^]^ The required time was far longer than anticipated: over 1 day for 25 mL (upright T75 flask) and over 5 days for 500 mL (**Figure** **1**A). The type of culture vessel or tube used did not change conditioning time for 25 or 50 mL of media. To understand media reoxygenation kinetics, 500 mL of 1% O_2_ medium was removed from a tri‐gas incubator and aliquoted into different culture vessels containing O_2_ sensors. By the time the media reached the culture vessels, the O_2_ concentration was >6% O_2_ (Figure 1B). Depending on the surface area of the medium in different culture vessels, the medium reached 10% O_2_ within seconds to 10 min. These results indicate that 1% O_2_ media conditioning requires surprisingly long periods – more than 5 days for large volumes. Furthermore, 1% O_2_ media is rapidly reoxygenated when removed from controlled O_2_ atmospheric environments, indicating that portable chambers and tri‐gas incubators are not suitable to condition media.

### 2.2 Cell Density, Medium Volume, and Culture Vessel Type Influence HIF Stabilization Kinetics

We investigated how cell density, medium volume, and culture vessel type influence the time it takes normoxic cultures to reach 1% O_2_. Non‐invasive optical sensor spots were used to measure pericellular O_2_ tension.^[^ ^47^, ^48^ ^]^ For MCF7 breast cancer cultures, all three parameters influenced the time to 1% O_2_, ranging from 1–14 h (Figure 1C,D). Cell density had the largest effect, suggesting that cellular O_2_ consumption plays a key role in the induction of hypoxia in vitro.

Next, we explored whether medium volume influenced cellular hypoxia kinetics. MCF7 cultures in 60 mm dishes containing either 5 mL or 15 mL of media were placed inside a 1% O_2_ incubator. Cellular hypoxia was evaluated using a hypoxia‐responsive fluorescent dye (Image‐iT Hypoxia) for 12 h. As expected, the cells in the 5 mL condition reached a maximum cellular fluorescence sooner than the 15 mL cultures: 4 versus 10 h (Figure 1E; Figure S1, Supporting Information).

To understand if cell density or culture vessel type influenced HIF stabilization in 1% O_2_ culture, MCF7 and MDA‐MB‐231 HIF reporter cell lines^[^ ^33^ ^]^ were cultured at two cell densities for 4 days and the percentage of GFP‐positive cells was determined using fluorescent microscopy. After 3 days, 2.2% of cells plated at the lower cell density were GFP‐positive whereas 22.9% of cells plated at a higher density were GFP‐positive (Figure 1F; Figure S2, Supporting Information). We then tested whether different culture vessel types would induce a similar effect for MDA‐MB‐231 reporter cells. Consistent with our findings in Figure 1C, T25 flask cultures had a higher percentage of GFP‐positive cells after 4 and 5 days compared to 24‐well cultures (Figure 1G). Collectively, we have demonstrated that cell density, medium volume, and culture vessel type, parameters that vary between experiments and are not reported, greatly influence cellular hypoxia and HIF stabilization kinetics. These results suggest that consistent HIF stabilization kinetics in 1% O_2_ culture can only be obtained by using a workstation and conditioned media.

### 2.3 Cellular O_2_ Consumption can Induce Anoxia in Both 5% and 1% O_2_ Culture

**Figure 2:**
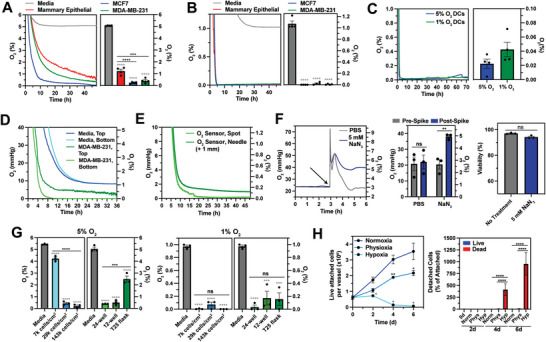
Cellular O_2_ consumption regularly induces anoxia (0% O_2_) in both physioxic (5% O_2_) and hypoxic (1% O_2_) culture. A,B) O_2_ profiles (left) and average O_2_ tension values (right) of media, human mammary epithelial, MCF7, or MDA‐MB‐231 cultures seeded onto a 24‐well plate in a 5% O_2_ (A) or 1% O_2_ (B) incubator for 72 h. C) O_2_ profiles (left) and average O_2_ tension values (right) of human dendritic cell (DC) 96‐well cultures in a 5% or 1% O_2_ incubator for 72 h. D) O_2_ kinetics of media (green) or MDA‐MBA‐231 cultures (blue) of the top (dark) or bottom (light) of the well in a 1% O_2_ incubator. E) O_2_ profiles of MDA‐MB‐231 cultures were measured at the bottom of the well (spot) or 1mm above the bottom of the well (needle) in a 1% O_2_ incubator. F) Effect of sodium azide (NaN_3_) on MCF7 pericellular O_2_ tension and cytotoxicity. O_2_ profiles (left) and average O_2_ tension values (middle) of MCF7 cultures spiked with either 5 mM NaN_3_ or PBS in a 1% O_2_ incubator. MCF7 cell viability after ± incubation with 5 mm NaN_3_ for 6 h in a 1% O_2_ incubator (right). G) Average O_2_ tension values of media and MCF7 cultures with different cell densities and culture vessel types in a 5% O_2_ (left) or 1% O_2_ incubator (right) for 72 h. H) Live, attached (left), and detached (right) cell counts for MCF7 cultures in an 18.6%, 5%, or 1% O_2_ incubator for 6 days. Data were analyzed with two‐tailed *t*‐test (F) or ANOVA and Dunnett's (A‐B) or Tukey's (C, F, G, H) corrections. N = 3–4 biological replicates per condition. Colored ^*^ indicates comparison to the control.

After discovering that cellular O_2_ consumption drives the induction of hypoxia in 1% O_2_ incubators, we hypothesized that it would also influence the pericellular O_2_ tension. Furthermore, we speculated that various cell types, each with differing rates of cellular O_2_ consumption, would influence this phenomenon. To test this, sub‐confluent (21000 cells cm^−2^) MCF7, MDA‐MB‐231, and primary human mammary epithelial cells were cultured in physioxic (5% O_2_) and hypoxic (1% O_2_) conditions for 72 h, and the pericellular O_2_ concentration was measured. Cell‐free media O_2_ tensions matched the incubator setpoints, indicating that the O_2_ sensor spots were accurately recording and the incubator O_2_ sensors were calibrated (**Figure** **2**A,B). In physioxia, the pericellular O_2_ was strikingly low – below 1.5% O_2_ for all three cell lines, and MCF7 cultures were anoxic (anoxia defined as <0.5% O_2_^[^ ^49^ ^]^). In hypoxia, the pericellular O_2_ of all cultures became anoxic (0% O_2_) within 5 h. Next, we investigated whether this effect also occurred in suspension culture. Activated human dendritic cells (DCs) were cultured in physioxia or hypoxia for 72 h. Both cultures were immediately and sustainably anoxic (0% O_2_) (Figure 2C).

If cellular O_2_ consumption did indeed affect pericellular O_2_ tension, there would be an axial O_2_ gradient in these cultures. To test this hypothesis, O_2_ tension at the media‐gas interface of MDA‐MB‐231 cultured at 1% O_2_ was measured using needle O_2_ microsensors (top) and pericellular O_2_ tensions were measured using sensor spots (bottom). Figure 2D illustrates that in these cultures, there is a gradient of 0.4–0% O_2_ from the top to the bottom of the well after 36 h. In wells without cells, both the top and bottom of the well reached 1% O_2_ as expected. To validate O_2_ sensor spot readings, needle microsensors were placed 1 mm above sensor spots in 1% O_2_ MDA‐MB‐231 cultures using a micromanipulator. Sensor spots recorded 0% O_2_ and needles recorded 0.1% O_2_, indicating that the two probes were in good agreement (Figure 2E).

To further validate the role of cellular O_2_ consumption in pericellular O_2_ tension, we tested whether inhibiting oxidative phosphorylation would ablate axial gradients. Upon addition of sodium azide (NaN_3_, complex IV inhibitor), the pericellular O_2_ tension of MCF7 cultures rapidly rose from 3% O_2_ to the incubator setpoint of 5% O_2_, whereas PBS (vehicle) spiked cultures returned to 3% O_2_ with continued incubation (Figure 2F, left panel). To confirm that this effect was not due to NaN_3_ cytotoxicity, we confirmed that there was no significant change in MCF7 cell viability when incubated with NaN_3_ at 5% O_2_ for 6 h (Figure 2F, right panel).

We next explored how cell density and culture vessel type influenced the gradient between atmospheric and pericellular O_2_ tensions. In physioxic MCF7 cultures, cell densities of 7000, 29000, and 143000 cells cm^−2^ in a 12‐well plate induced pericellular O_2_ tensions of 4.2%, 0.5%, and 0.1% O_2_, respectively. Different culture vessels (24‐well plate, 12‐well plate, and T25 flask) also influenced MCF7 tensions, ranging from 0.4–2.5% O_2_ (Figure 2G, left panel). In hypoxia, neither cell density nor culture vessel type change pericellular tensions in MCF7 cultures: all were anoxic (<0.2% O_2_) (Figure 2G, right panel).

After determining that all hypoxic MCF7 cultures tested were anoxic, we sought to understand how pericellular anoxia affected cell viability. MCF7 cells were cultured in normoxia, physioxia, or hypoxia for 6 days and cell proliferation and viability were evaluated. Cells cultured in normoxia and physioxia proliferated throughout the 6‐day period (Figure 2H, left panel). However, cells cultured in hypoxia did not proliferate, and the majority were dead and detached after 4 days (Figure 2H, right panel).

Collectively, these experiments show that cellular O_2_ consumption drives pericellular O_2_ far below the incubator setpoint, inducing anoxia in both physioxic and hypoxic MCF7, MDA‐MBA‐231, and human DC cultures. Furthermore, in physioxic MCF7 culture, pericellular O_2_ tension is highly dependent on cell culture parameters, ranging from 0.1–4.2% O_2_.

### 2.4 Setting the Incubator to Physioxia Mimics Ischemia‐Reperfusion Injury in Hepatocyte Culture

**Figure 3:**
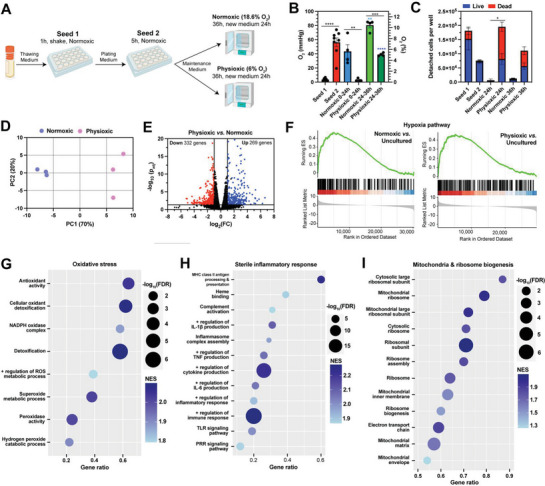
Setting the incubator to physiological O_2_ conditions mimics ischemia‐reperfusion injury in human hepatocyte culture. A) Schematic of the primary human hepatocyte culture. RNA‐seq was performed on uncultured hepatocytes and cells cultured in normoxic (18.6% O_2_) or physioxic (6% O_2_) incubator after 36 h. The physioxic setpoint was chosen based on the O_2_ tension found in the human liver parenchyma.^[^ ^17^ ^]^ B,C) Average O_2_ tension values (B) and detached cells per well (C) during each step of the culturing process. D) Principal component analysis (PCA) of the RNA‐seq results for normoxic and physioxic cultured hepatocytes. E) Volcano plot indicating upregulated (blue) and downregulated (red) genes for physioxic vs. normoxic cultured hepatocytes (p_adj_ < 0.05 and |log_2_FC| ≥ 1). F) Hypoxia gene set enrichment analysis (GSEA) from the Hallmark database for normoxic vs. uncultured (left) and physioxic vs. uncultured (right). G–I) Enriched pathways from the Gene Ontology (GO) database for physioxic vs. normoxic samples associated with oxidative stress (G), sterile inflammatory response (H), and mitochondrial and ribosomal biogenesis (I). Data were analyzed with ANOVA and Tukey's correction (B,C). N = 3 biological samples per condition for RNA seq analysis.

We next explored how the difference between the incubator setpoint and pericellular O_2_ tension can impact the physiological relevance of cell culture models. Because of their high O_2_ consumption rate^[^ ^17^, ^50^ ^]^ and widespread use as an in vitro drug metabolism model,^[^ ^51^ ^]^ primary human hepatocytes were used for these studies. Hepatocytes were seeded and cultured in either normoxic (18.6% O_2_) or physioxic (6% O_2_^[^ ^17^ ^]^) conditions for 36 h (**Figure** **3**A). The physioxic setpoint was selected based on the O_2_ tension observed in human liver parenchyma.^[^ ^17^ ^]^

During the first initial seeding step, which was conducted in normoxia, hepatocytes were anoxic (0.5% O_2_) (Figure 3B; Figure S3, Supporting Information). During the first 24 h of culture, hepatocytes cultured in normoxia were physioxic (5.6% O_2_) and hepatocytes cultured in physioxia were anoxic (0.4% O_2_). Unexpectedly, when media was exchanged after 24 h, both types of cultures underwent reoxygenation for the duration of the experiment. Figure 3C illustrates that the pericellular anoxia experienced by hepatocytes cultured in physioxia increased the number of detached cells after 24 h, the majority of which were dead.

To investigate how the pericellular O_2_ tension influenced hepatocyte physiology, we assessed gene expression by RNA‐seq of uncultured hepatocytes and hepatocytes cultured in normoxia or physioxia after 36 h. Principal component analysis (PCA) of the transcriptome shows clustering of replicates by O_2_ tension, as expected (Figure 3D). Differential gene expression analysis (physioxic versus normoxic) found 269 upregulated and 332 downregulated genes (Figure 3E). Gene set enrichment analysis (GSEA) revealed an upregulation in hypoxia‐associated genes in normoxic and physioxic cultured cells compared to uncultured hepatocytes (Figure 3F), indicating that the 1‐h anoxic seeding step induced a hypoxic response. Examining 195 genes within the Hallmark Hypoxia gene set reveals that the hypoxic response was similar between physioxic and normoxic cultures (Figure S4, Supporting Information). In addition, GSEA suggests that hepatocytes cultured in physioxia mounted an oxidative stress response (Figure 3G). Corroborating the higher cell death and detachment, physioxic cultured hepatocytes also had an upregulation in IL‐1β production, TNF production, TLR signaling, and PRR signaling pathways, indicating a sterile inflammatory response (Figure 3H). Lastly, hepatocytes in physioxia had upregulated mitochondrial and ribosomal biogenesis pathways (Figure 3I). Taken together, these results indicate that setting the incubator to physiological O_2_ conditions mimics ischemia‐reperfusion injury in hepatocytes due to their cellular O_2_ consumption.

### 2.5 Developing a Reaction‐Diffusion Model to Predict Pericellular O_2_ Tension in Cell Cultures

Measuring pericellular O_2_ tension for every O_2_‐controlled cell culture experiment would be cumbersome and expensive. We hypothesized that a computational model could predict pericellular tension a priori, given cell density, O_2_ consumption rate, culture vessel type and medium volume. Such a tool would reduce the need for experimental measurements.

**Figure 4:**
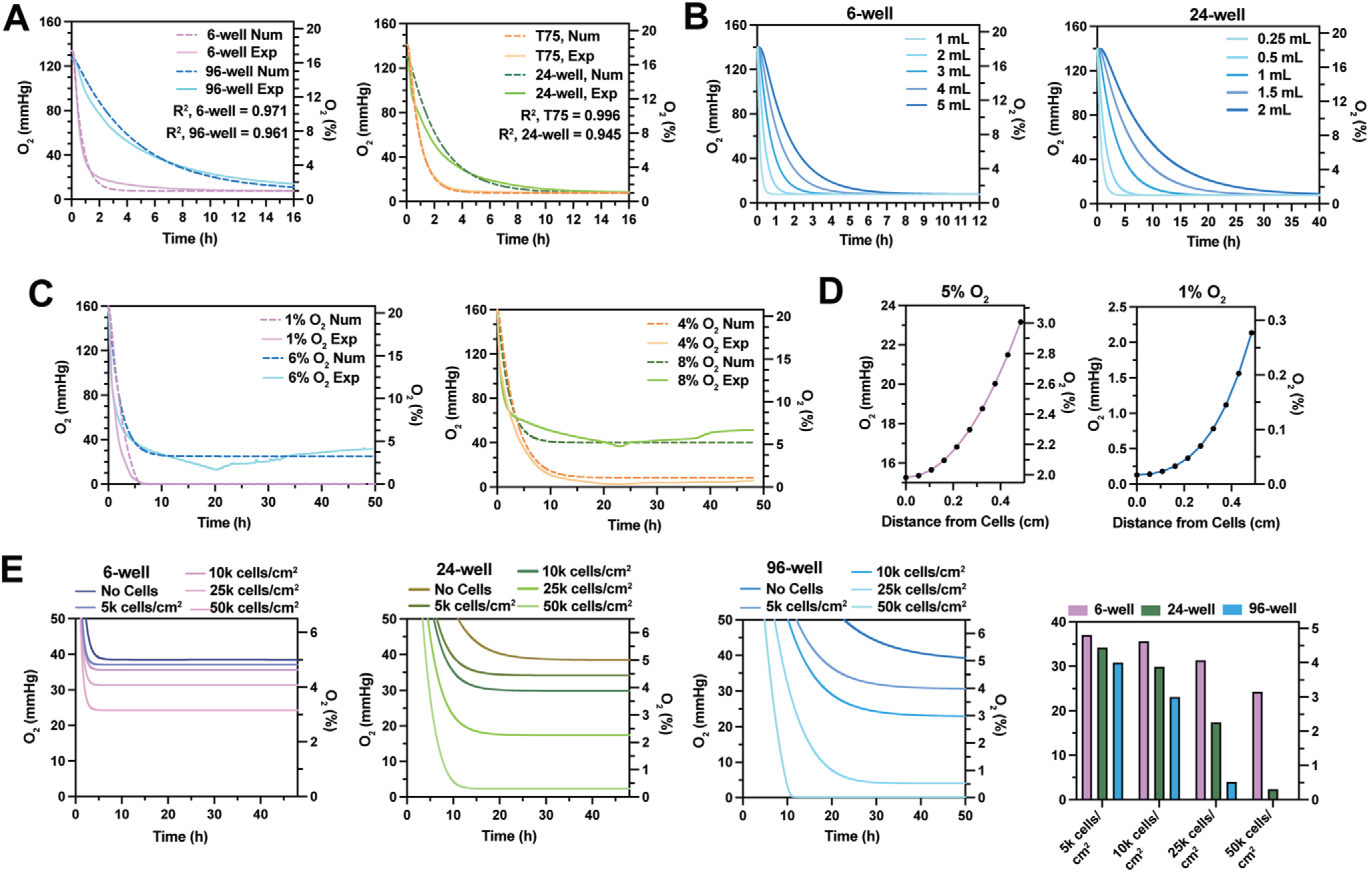
Developing a reaction‐diffusion model to predict pericellular O_2_ tension in cell cultures. A) Numerical (num) (dashed) and experimental (exp) (solid) O_2_ kinetics of normoxic media (EMEM + 10% FBS + 1% P/S) in different culture vessels placed in a 1% O_2_ incubator. B) Diffusion model predictions for different volumes of normoxic media in a 6‐well plate (left) or a 24‐well plate (right) placed in a 1% O_2_ incubator. C) Numerical and experimental O_2_ kinetics of MDA‐MB‐231 cultures seeded in a 24‐well plate placed in a 1%, 4%, 6%, or 8% O_2_ incubator for 48 h. These setpoints were selected to test and validate the reaction‐diffusion model. D) Reaction‐diffusion model predictions of the gradient within MDA‐MB‐231 cultures seeded in a 24‐well plate in a 5% O_2_ (left) or 1% O_2_ (right) incubator. E) Reaction‐diffusion model predictions of MDA‐MB‐231 cells cultured at different cell densities and culture vessel types in a 5% O_2_ incubator. N = 4 biological replicates per condition for experimental data.

We first examined whether the unsteady state diffusion equation^[^ ^40^, ^41^ ^]^ could describe O_2_ transfer kinetics between cell culture medium and incubator gas phases. Coefficient of determination (R^2^) values suggested that experimental and numerical values that describe the O_2_ transfer between normoxic media and 1% O_2_ gas phase in different culture vessels were in good agreement (**Figure** **4**A). The diffusion model also predicted the equilibration of normoxic media in a 5% O_2_ incubator (Figure S5A, Supporting Information). In addition, the diffusion model's analytical solution was also comparable to numerical solutions for different culture vessel types (Figure S5B, Supporting Information).

After validating the diffusion model, we applied it to investigate the dependency of O_2_ transfer kinetics on medium volume in a 24‐well plate and 6‐well plate. Figure 4B shows that although commonly used medium volumes for 24‐well and 6‐well plates modestly impact the time to 1% O_2_, there is a substantial difference in kinetics between 24‐well and 6‐well plate wells. 6‐well plate volumes of media require 0.5–12 h to reach 1% O_2_, whereas 24‐well plate requires 2.5–40 h. Importantly, the rate of equilibration for the same medium volumes (1 mL and 2 mL) is substantially lower in 24‐well plate than 6‐well plate wells. These findings indicate that the gas phase–pericellular O_2_ differential increases as culture vessel surface area decreases (Figure 2G) because of a decrease in O_2_ transfer rates.

Next, we developed a reaction‐diffusion model to describe pericellular O_2_ tension in cell cultures within O_2_‐controlled environments. Michaelis–Menten kinetics were used to model cellular O_2_ consumption.^[^ ^43^ ^]^ This model predicts pericellular O_2_ tension values for MDA‐MB‐231 cultured at 1%, 4%, 6%, and 8% O_2_ with reasonable Michael–Menten parameters (V_max_ = 450 amol cell^−1^ sec^−1^ and K_m_ = 1 µm)^[^ ^43^ ^]^ (Figure 4C). These O_2_ setpoints were chosen to validate the reaction‐diffusion model. For further validation, we tested whether the model predicted axial O_2_ gradients like those experimentally determined in Figure 2D. The model predicts gradients of 2.0–3.0% O_2_ and 0–0.3% O_2_ for 30000 cells cm^−2^ MDA‐MB‐231 cultures in physioxia and hypoxia, respectively (Figure 4D), in good agreement with experimental data.

Using the reaction‐diffusion model, we next examined the influence of cell density on pericellular O_2_ tension in different culture vessel types in physioxia. The model predicts that the highest cell density will have a modest influence on pericellular tension in 6‐well plate cultures (3.2% O_2_), but it will induce anoxia in 24‐well (0.4% O_2_) and 96‐well plate cultures (0% O_2_) (Figure 4E). Lastly, we investigated the influence of cellular O_2_ consumption (V_max_) in hypoxia (Figure S5C, Supporting Information). Both cell density and O_2_ consumption predictions suggest that the smaller the culture vessel surface area, the greater the impact of cellular O_2_ consumption on pericellular O_2_ tension. This effect is due to the decreasing media surface area to height as culture vessel size decreases (Figure S5D, Supporting Information). Taken together, we establish that a reaction‐diffusion model can predict pericellular O_2_ tension in O_2_‐controlled cell culture. Furthermore, using the model, we discovered that the effect of cellular consumption on pericellular O_2_ tension is highly dependent on the culture vessel type, increasing as culture vessel size decreases.

### 2.6 Pericellular Anoxia Induces Stronger Metabolic Reprogramming Compared to Pericellular Hypoxia in MCF7 Cells

The studies presented thus far demonstrate that standard hypoxic cell culture (1% O_2_) routinely induces anoxia due to cellular O_2_ consumption. Because anoxia is not physiologically relevant in vivo, we asked whether anoxia is suitable to model hypoxia. To explore this concept, we controlled pericellular O_2_ tension to investigate cancer cell responses to pericellular hypoxia (1–2% O_2_) versus pericellular anoxia (0–0.5% O_2_).

**Figure 5:**
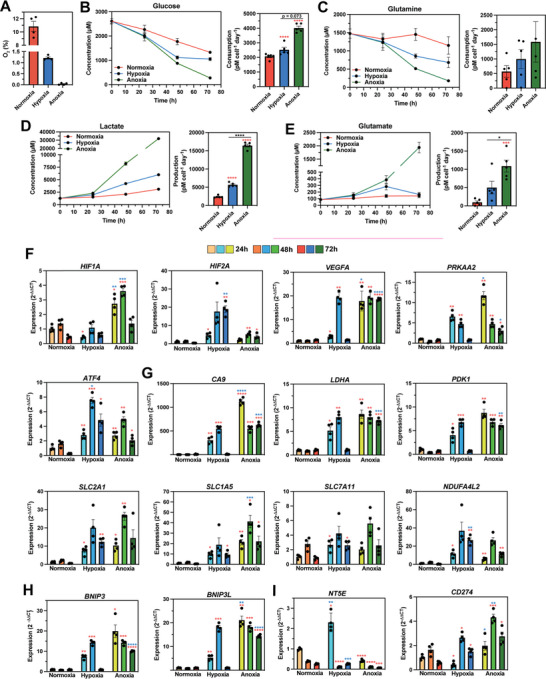
Pericellular anoxia induces stronger metabolic reprogramming and distinct transcriptional *HIFA* and HRE‐controlled gene responses compared to pericellular hypoxia in MCF7 cells. A) Average pericellular O_2_ tensions in MCF7 cultures placed in 18.6%, 4.5%, and 1% O_2_ incubators for 72 h. B–E) Extracellular concentrations of glucose B), glutamine C), lactate D), and glutamate E) in different O_2_ tensions for 72 h. Normalized metabolite concentrations over time (left) and metabolite consumption or production rates for 72 h (right). F–I) Gene expression levels of genes associated with the hypoxic response F), metabolic reprogramming (G), autophagy H), and immunosuppression I) in different O_2_ tensions for 72 h. Data were analyzed using ANOVA with Tukey's correction. N = 4 biological samples per condition. Color code for asterisk (*): Red colored * indicates a comparison to control (normoxia), and blue colored * indicates a comparison between hypoxia and anoxia.

First, we examined MCF7 metabolic reprogramming in response to different pericellular O_2_ tensions. Expected metabolic changes in response to hypoxia include an increase in (*i*) glucose consumption due to increased uptake and glycolytic flux, (*ii*) extracellular lactate from decreased TCA cycle flux and increased lactate transport, (*iii*) glutamine uptake to replenish TCA cycle intermediates for lipid metabolism, and (*iv*) extracellular glutamate secretion, which promotes cancer cell proliferation.^[^ ^46^, ^49^, ^52^, ^53^, ^54^, ^55^ ^]^ MCF7 cells were cultured for 72 h in 18.6%, 3.5–4.5%, and 1% O_2_ incubators, resulting in supraphysiologic (10.8% O_2_), hypoxic (1.2% O_2_), and anoxic (0% O_2_) pericellular tensions, respectively (**Figure** **5**A). To maintain pericellular hypoxia, the incubator's gas phase was adjusted from 4% to 3.5% O_2_ at 27 h and from 3.5% to 4.5% O_2_ at 53 h (Figure S6A, Supporting Information). These changes in gas phase O_2_ tension led to a rapid change in pericellular O_2_ tension. Additionally, a constant 1% O_2_ gas phase was sustained to maintain pericellular anoxia throughout the entire time course (Figure S6B, Supporting Information). Consumption (glucose and glutamine) and production (lactate and glutamate) rates trended higher with decreasing pericellular O_2_ tension over the 72‐h time course (Figure 5B–E). For example, lactate and glutamate production was 2.2‐ and 1.6‐fold higher for cells in anoxia compared to hypoxia, respectively. Only the anoxic MCF7 cultures exhibited increased rates after 24  h, whereas hypoxic and normoxic cultures maintained constant rates. These findings suggest that for MCF7 cells, anoxia induces a stronger metabolic reprogramming response than hypoxia does.

### 2.7 Pericellular Hypoxia Versus Anoxia Induces Distinct Transcriptional HIFα and HRE Gene Responses in MCF7 Cells

We examined MCF7 transcriptional responses to low O_2_ tensions at 24, 48, and 72 h, including genes associated with (*i*) the hypoxic response (e.g., hypoxia inducible factor 1 subunit alpha (*HIF1A*), hypoxia inducible factor 2 subunit alpha (*HIF2A*), vascular endothelial growth factor A (*VEGFA*), protein kinase AMP‐activated catalytic subunit alpha 2 (*PRKAA2*), and activating transcription factor 4 (*ATF4*)); (*ii*) metabolic reprogramming (e.g., carbonic anhydrase 9 (*CA9*), lactate dehydrogenase A (*LDHA*), pyruvate dehydrogenase kinase 1 (*PDK1*), solute carrier family 2 member 1 (*SLC2A1*, GLUT1), solute carrier family 1 member 5 (*SLC1A5*), solute carrier family 7 member 11 (*SLC7A11*, xCT), and NADH dehydrogenase 1 alpha subcomplex, 4‐like 2 (*NDUFA4L2*)); (*iii*) mitophagy (e.g., Bcl‐2 interacting protein 3 (*BNIP3*) and Bcl‐2 interacting protein 3 like (*BNIP3L*)); and (*iv*) the immunosuppressive tumor microenvironment (TME) (e.g., cluster of differentiation 274 (*CD274*, PD‐L1) and 5′‐nucleotidase ecto (*NT5E*, CD73^[^ ^56^, ^57^ ^]^) (Figure 5F–I). Nearly all of these genes are direct HIF targets (i.e., hypoxia‐responsive element (HRE)‐controlled genes), excluding *PRKAA2*, *ATF4*, and *SLC1A5*.^[^ ^37^, ^49^ ^]^

In anoxia, *VEGFA*, *CA9*, *PDK1*, *BNIP3*, and *BNIP3L* show elevated expression compared to normoxia throughout the time course. However, in hypoxia, these genes steadily increased expression and peaked after 48 h, followed by a drop to normoxic expression levels after 72 h. Interestingly, *HIFA* expression was different in hypoxia and anoxia: *HIF1A* had higher expression in anoxia (Six and threefold after 24 and 48 h, respectively) and *HIF2A* had higher expression in hypoxia (fivefold after 72 h) (Figure 5F). In addition, MCF7 had higher expression of *PRKAA2* (AMPK α2 subunit) in anoxia (two and fourfold after 24 and 72 h, respectively), suggesting lower ATP availability in anoxia.^[^ ^58^ ^]^ Interestingly, hypoxia induced a twofold higher expression of *ATF4* after 48 h than did anoxia, indicating a stronger integrated stress response in hypoxia.^[^ ^59^ ^]^ Glycolytic genes had higher expression in anoxia (*LDHA* sevenfold and *PDK1* ninefold after 72 h). *SLC2A1* (GLUT1) expression levels also trended higher in anoxia throughout the time course. *SLC1A5* expression levels were twofold higher in anoxia after 48 h. Overall, these results support the metabolic profiles in Figure 5B–E.

*BNIP3* and *BNIP3L* expression levels were higher in anoxia after 72 h, suggesting an upregulation in mitophagy in anoxia.^[^ ^49^ ^]^ Lastly, in the context of immunosuppression, hypoxia induced a sixfold higher expression of *NT5E* (CD73, extracellular AMP to adenosine conversion^[^ ^56^, ^57^ ^]^) after 24 h. Anoxia induces twofold higher expression of *CD274* (PD‐L1) after 48 h. Overall, these results indicate that hypoxia and anoxia induce distinct expression profiles in both *HIFA* and HRE‐responsive genes in MCF7.

### 2.8 Proteomic Characterization of the Temporal Differences between Pericellular Hypoxic and Anoxic Responses in 4T1 Cells

After looking at transcriptional responses to hypoxia and anoxia, we aimed to better understand changes in protein expression in response to low O_2_ tensions. To this end, we applied plexDIA^[^ ^60^ ^]^ to understand how the proteome changes in response to hypoxia and anoxia in a murine triple‐negative breast cancer (TNBC) cell line (4T1). PCA of the proteome shows clustering by O_2_ tension and by day (Figure S7A, Supporting Information). Furthermore, hypoxic and anoxic samples shift away from normoxic samples along PC1 over time, indicating continuing changes during low O_2_ responses.

For the hypoxic response, differential protein abundance analysis indicates no significantly upregulated or downregulated proteins after 1 day of culture, with the maximum response occurring after 3 days. On the other hand, the anoxic response had 50 upregulated and 5 downregulated proteins after day 1, and the response peaked after only 2 days. The number of changing proteins was higher in anoxia than hypoxia for all three days (Figure S7B, Supporting Information).

Protein set enrichment analysis (PSEA) was performed to compare each low O_2_ response between days. In agreement with the differential protein abundance analysis, PSEA suggests that the anoxic response is faster and peaks by day 2: most of the changes occur for 2 days versus 1 day (Figure S7C, Supporting Information). For both O_2_ conditions, hypoxia‐associated pathways are upregulated throughout the time course, including hypoxia, glycolysis, cholesterol homeostasis, fatty acid metabolism, and epithelial‐to‐mesenchymal transition (EMT). Interestingly, Myc targets were downregulated in both tensions for all days, which may contribute to the reduction in proliferation at low O_2_ tensions.^[^ ^61^ ^]^ Overall, this temporal characterization of the proteome suggests that the response to anoxia is stronger and faster than the response to hypoxia in 4T1 cells.

### 2.9 Characterizing Proteomic Differences in Metabolic Reprogramming between Pericellular Hypoxic and Anoxic Responses in 4T1 Cells

**Figure 6:**
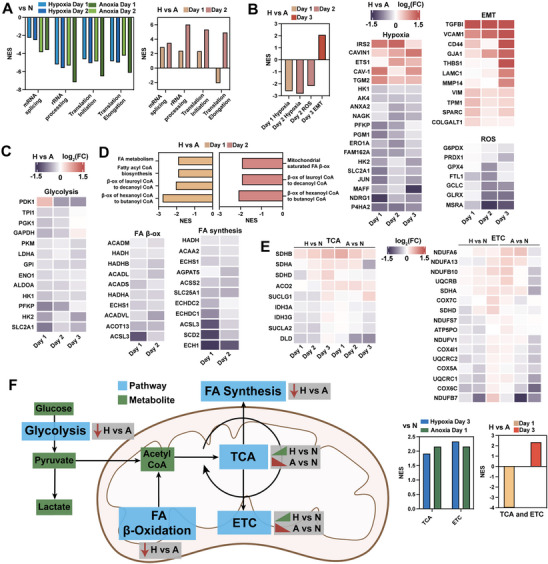
Proteomic characterization of pericellular hypoxic and anoxic metabolic reprogramming in 4T1 cells. A) Protein set enrichment analysis (PSEA) using the Hallmark database of mRNA processing and protein translation pathways for Hypoxia (H) vs. Normoxia (N), Anoxia (A) vs. Normoxia, and Hypoxia vs. Anoxia. B) PSEA using the Hallmark database of hypoxic response pathways (left). Heat maps of selected proteins in Hallmark hypoxia, epithelial to mesenchymal transition (EMT), and reactive O_2_ species (ROS) pathways (right) (Hypoxia vs. Anoxia). C) Heat map of glycolytic and hypoxic response proteins. D) PSEA using Reactome database for fatty acid (FA) metabolism pathways for Hypoxia vs. Anoxia (top). Heat maps of selected proteins in fatty acid β‐oxidation and synthesis Reactome pathways (bottom). E) Heat maps of selected proteins from Tricarboxylic acid (TCA) cycle and electron transport chain (ETC) processes for Hypoxia vs. Normoxia and Anoxia vs. Normoxia. PSEA using the Reactome database of TCA and ETC pathways for Hypoxia vs. Normoxia, Anoxia vs. Normoxia (left), and Hypoxia vs. Anoxia (right). F) Illustration of findings between normoxic, hypoxic, and anoxic 4T1 responses. NES = normalized enrichment score.

To further characterize low O_2_ responses in 4T1 cells, we explored protein synthesis, hypoxic responses, and metabolic reprogramming at the pathway and protein level. As expected, RNA processing and protein translation pathways were downregulated in hypoxia and anoxia compared to nomoxia (**Figure** **6**A). Most of these pathways were upregulated in hypoxia compared to anoxia, suggesting downregulation as a function of O_2_ tension. Surprisingly, translation elongation was upregulated in anoxia compared to hypoxia after 1 day of culture, suggesting distinct regulation in the acute anoxic response.

Next, we looked at pathways associated with the hypoxic response and discovered that hypoxia and reactive O_2_ species (ROS) pathways were upregulated in anoxia, and EMT was upregulated in hypoxia (Figure 6B). Proteins associated with hypoxia‐induced stress responses showed higher abundance in anoxia for all three days. In addition, proteins associated with tumor progression, invasion, and metastasis, were upregulated in hypoxia for the duration of the experiment.

Figure 6C illustrates that 9 out of 10 enzymes involved in glycolysis are more abundant in anoxia compared to hypoxia. In addition, proteins involved in hypoxia‐mediated metabolic reprogramming show increased abundance in anoxia. We also examined fatty acid β‐oxidation and synthesis, which are downregulated and upregulated in hypoxia respectively.^[^ ^37^, ^49^ ^]^ Interestingly, PSEA suggests that both of these processes are downregulated in anoxia compared to hypoxia for the first 2 days of culture (Figure 6D). Most enzymes involved in mitochondrial acyl‐CoA to acetyl‐CoA conversion (i.e., fatty acid β‐oxidation) were upregulated in anoxia.

Finally, we examined oxidative phosphorylation, which is expected to decrease in low O_2_ tensions.^[^ ^37^, ^49^ ^]^ Unexpectedly, for hypoxia versus anoxia, the tricarboxylic acid (TCA) cycle and electron transport chain (ETC) processes were downregulated after 1 day of culture and upregulated after 3 days of culture (Figure 6E). Clustering of TCA and ETC enzymes suggests increasing oxidative phosphorylation activity in hypoxia, yet decreasing activity in anoxia (compared to normoxia). Taken together, these results suggest that 4T1 cells have distinct hypoxic and anoxic metabolic responses (Figure 6F).

## 3 Discussion

Despite the widespread use of O_2_‐controlling chambers in hypoxia‐related research, studies quantifying pericellular O_2_ concentrations and their impact on cellular response are surprisingly lacking. In the current study, we discovered vast differences between incubator setpoints and pericellular O_2_ tensions in every cell type tested.

Our results highlight a major challenge with portable chambers and tri‐gas incubators: cultures cannot be conditioned to start at the desired O_2_ tension due to rapid reoxygenation of media upon exposure to normoxia. Without media conditioning, MCF7 cultures can take 1–14 h to reach 1% O_2_ depending on the experimental set‐up. Not only does this time difference introduce significant variability between experiments, but it also suggests that shorter hypoxic experiments may not even reach hypoxia. We also show that changing cell culture parameters induce a sustained difference of HIF stabilization kinetics for at least 5 days for two different cell lines. Unless a hypoxic workstation with preconditioned media is used, pericellular O_2_ tension must be determined to report accurate O_2_‐controlled incubation times.

Challenging the belief that incubators accurately control O_2_ for cell cultures, we discovered that pericellular anoxic tensions are common in both physioxic (5% O_2_) and hypoxic (1% O_2_) conditions due to cellular O_2_ consumption. This effect occurred in both primary human adherent and suspension cultures, with commonly used cell densities, medium volumes, and culture vessel types. Furthermore, our results suggest that physioxic cultures are routinely hypoxic. O_2_ tension in physioxia can vary greatly (0.1–4.5% O_2_) depending on experimental set‐up, engendering reproducibility concerns. Furthermore, it is evident that the type of cell impacts pericellular O_2_ tension in physioxic culture, with primary cells exhibiting higher tension compared to cancer cell lines from the same tissue. These findings are a major concern for physioxic culture of stem cell expansion and differentiation,^[^ ^62^, ^63^, ^64^ ^]^ and controlling pericellular O_2_ tension may improve our understanding of these processes. Future studies will examine how different cell types and proliferation rates influence pericellular O_2_ tension.

Our pericellular O_2_ tension results for primary human hepatocyte cultures, a prominent model in drug metabolism, underpin how the incubator setpoint is a poor indicator of the O_2_ cells experience. Hepatocytes cultured in physioxia experienced anoxia for 24 h, followed by a rapid reoxygenation upon media exchange. RNA‐seq results indicate that O_2_ fluctuations in these conditions induce an upregulation in cellular responses to mitochondrial and NADPH oxidase ROS production (e.g., superoxide, hydrogen peroxide).^[^ ^65^, ^66^ ^]^ Oxidative stress increased cell death via upregulation of TNF (apoptosis) and IL‐1β (pyroptosis) production.^[^ ^67^, ^68^ ^]^ Damage‐associated molecular patterns (DAMPs) released by dying cells activate the toll‐like receptor (TLR) and pattern recognition receptor (PRR) pathways, inducing key signatures of a sterile inflammatory response: complement activation, inflammasome complex assembly, MHC class II upregulation, and IL‐6 production.^[^ ^69^, ^70^ ^]^ During reoxygenation, hepatocytes increased mitochondria and ribosome biogenesis to meet ATP and protein translation demands as the cells recovered from hypoxic exposure.^[^ ^49^ ^]^ Ultimately, these results indicate that physioxic culture of hepatocytes drives a cellular response mimicking liver ischemia‐reperfusion injury, a major risk factor in graft dysfunction in liver transplantation.^[^ ^68^ ^]^ Unexpectedly, our O_2_ measurements and RNA‐seq results both suggest that hepatocytes, when cultured in normoxia, experienced hypoxia during the first initial seeding step. This observation is likely attributable to the high O_2_ consumption rate of hepatocytes,^[^ ^50^ ^]^ a high cell density (0.5 × 10^6^ cells per 24‐well plate well), and adaptation from a freeze‐thaw cycle to in vitro conditions. This finding underscores the disparity between pericellular O_2_ tension and the surrounding O_2_ concentration.

We developed a reaction‐diffusion model, which accurately predicted pericellular O_2_ tensions of MDA‐MB‐231 cultures at various incubator setpoints. This novel tool can design O_2_‐controlled cell culture experiments, modulating parameters like the cell density, culture vessel type, or medium volume to achieve desired pericellular O_2_ concentrations. Our finding that the effect of cellular O_2_ consumption increases as culture vessel size decreases suggests that smaller vessels (e.g., 96‐well plates) should be avoided for O_2_‐controlled cell culture. This observation carries significant implications for immune cell culture, which is typically done at high cell densities in small culture vessels. Future iterations of the model should consider incorporating cell growth rates as a function of O_2_ tension. In addition, experiments to determine O_2_ consumption rates (V_max_) for different cell types are needed. Such studies may uncover O_2_ consumption trends applicable to most cell types.

Using the reaction‐diffusion model and manipulation of gas phase O_2_, we performed the first investigation into the relationship between pericellular O_2_ tension and biological response. We investigated the metabolic, transcriptomic, and translational responses to hypoxia (1–2% O_2_) and anoxia (0% O_2_) in two different breast cancer cell lines. Quantification of 14 genes associated with hypoxia (11 are direct HIF targets) and *HIF1A*/*HIF2A* suggest distinct transcriptional responses in hypoxia and anoxia. In anoxia, we found higher transcription in *HIF1A*, hypoxic markers (*VEGFA*, *CA9*), metabolism (*LDHA*, *PDK1*, *SLC1A5*), mitophagy (*BNIP3*, *BNIP3L*), and *CD274* (PD‐L1) compared to hypoxia. Conversely, *HIF2A*, *ATF4*, *NDUFA4L2*, and *NT5E* (CD73) was upregulated in hypoxia. The increase in *HIF1A* transcription in anoxia may occur through ROS‐induced PI3 kinase (PI3K) and protein kinase C (PKC) pathways,^[^ ^71^, ^72^ ^]^ since our proteomic analysis suggests that ROS production is higher in anoxia.

A previous study reported that maximum HIF1 DNA‐binding activity occurs at pericellular 0.5% O_2_ and sharply decreases as tension approaches 0% O_2_.^[^ ^73^ ^]^ This suggests that in anoxic culture, HIF is maximally stabilized as cultures approach anoxia (rather than in anoxia per se) inducing a strong HRE transcriptional response. Ultimately, 0.5% pericellular O_2_ may be the ideal setpoint for hypoxic cell culture, as long as tensions do not drop to anoxic levels. Future studies will explore the post‐translational modification of both HIF1 and HIF2, as well as downstream responses, under various low O_2_ tensions, ranging from 0% to 3% O_2_. These studies aim to provide a mechanistic understanding of how cells respond differently to these tensions.

To further understand low O_2_ tension responses, we performed an in‐depth characterization of the 4T1 proteome in response to low O_2_ tensions. This analysis suggests that the global translational response to anoxia is stronger and faster than the hypoxic response. Yet, the responses are distinct: we found an upregulation of EMT proteins in hypoxia and an increased ROS response in anoxia for 72 h of culture. EMT is a critical step in hypoxia‐driven metastasis^[^ ^74^ ^]^ and metastatic breast cancer represents the most advanced stage of the disease.^[^ ^75^ ^]^ Our findings suggest that mitochondrial dysfunction and ROS production are higher in anoxia than in hypoxia.^[^ ^76^ ^]^

There are several limitations to this study. First, we did not use a hypoxic workstation or conditioned media, indicating that our O_2_‐controlled cultures did not immediately reach the desired O_2_ tension. Second, we did not differentiate whether responses in different O_2_ tensions were due to HIF‐dependent or HIF‐independent mechanisms, nor did we provide a mechanistic understanding of the differences between hypoxic and anoxic responses. Lastly, we examined cancer cell responses to low O_2_ tensions after culturing them in supraphysiological O_2_ (i.e., normoxia), which could potentially influence the responses.

Ultimately, our exploration of breast cancer responses to low O_2_ tensions suggests that anoxia is not suitable to model hypoxia. This is fortified by the fact that the median O_2_ tension in breast tumors is 10 mmHg (1.3% O_2_).^[^ ^77^ ^]^ Equally important, our findings uncover that breast cancer cells respond non‐monotonically to low O_2_ since many aspects of the low O_2_ response are upregulated in hypoxia compared to anoxia. Future work will further explore these responses and relate them to hypoxic environments in vivo.

## 4 Conclusion

O_2_ is a critical factor for mammalian bioenergetic homeostasis and serves as a substrate for over 200 enzymatic reactions.^[^ ^12^ ^]^ O_2_‐controlled cell culture attempts to mimic O_2_ tensions that cells are exposed to in vivo and is, therefore, a critical tool for biological research. Herein, we report the discovery that the metric used to determine O_2_ concentration for in vitro cultures, the incubator setpoint, is a poor indicator of the O_2_ tension cells actually experience (i.e., pericellular O_2_ tension) due to cellular O_2_ consumption. Standard physioxic (5% O_2_) and hypoxic (1% O_2_) protocols routinely induce anoxia (0% O_2_). Furthermore, in physioxic culture, pericellular O_2_ tension is highly dependent on cell culture parameters, making reproducibility difficult. Highlighting the significance of these findings, we demonstrated that a key drug metabolism model, primary human hepatocytes, undergo an effect similar to ischemia‐reperfusion injury when cultured in physioxia. To address these challenges, we developed a reaction‐diffusion model that predicts pericellular O_2_ tension a priori. Using this tool, we controlled pericellular O_2_ tension in two breast cancer models to explore transcriptional and translational responses to hypoxia and anoxia. We discovered that breast cancer cells respond non‐monotonically to low O_2_. Overall, this work calls for a fundamental change to how O_2_‐controlled cell culture is performed and suggests that pericellular O_2_‐controlled cell culture is necessary to accurately model O_2_ tension.

## 5 Experimental Section

### In Vitro O_2_ Measurements

Adhesive optical O_2_ sensor spots (OXSP5‐ADH‐STER, PyroScience GmbH) were used to measure the O_2_ concentration of media and cell cultures as previously described.^[^ ^78^ ^]^ Sensors were placed on the culture vessel surface and a cable adapter (SPADBAS, PyroScience GmbH) was glued on the opposite side of the culture vessel (lined up with the sensor). Glue was allowed to dry overnight. Optical fiber cables (SPFIB‐BARE, PyroScience GmbH) were placed within the adapters and connected to a computer via a meter (FireSting O_2_, PyroScience GmbH). The 100% O_2_ calibration was performed with aerated Dulbecco's phosphate‐buffered saline (DPBS), and the 0% O_2_ calibration was performed using the factory calibration value. For cell culture experiments, cells were seeded in sensor‐containing culture vessels, and pericellular O_2_ was measured. A temperature probe (TDIP15, PyroScience GmbH) connected to the meter was placed inside the same incubator as the sensor‐containing culture vessels. To measure the O_2_ concentration at the top of the media or cell culture wells, needle‐like probes (OXROB10, PyroScience GmbH) were attached to a micromanipulator (MM33, PyroScience GmbH) and placed at the media–gas interface, such that the probes were submerged at the top layer of the media. Holes were drilled in plate lids to allow the probes to reach the media.

### Cell Culture

Mycoplasma‐free cell lines, MDA‐MB‐231 (ATCC HTB‐26), MCF7 (ATCC HTB‐22), 4T1 (ATCC CRL‐2539), and primary mammary epithelial (ATCC PCS‐600‐010) were obtained from the American Type Culture Collection (ATCC). MDA‐MB‐231, MCF7, and 4T1 were maintained in Leibovitz's L‐15 medium (Cytiva), Eagles’ Minimum Essential Medium (EMEM) with L‐glutamine (Quality Biological) and Dulbecco's Minimum Essential Medium (DMEM) (Corning), respectively, with 10% fetal bovine serum (FBS, Corning) and 1% penicillin/streptomycin (P/S, Invitrogen). Mammary epithelial cells were cultured in basal medium (ATC PCS‐600‐030) with cell growth kit (ATCC PCS‐600‐040). MCF7 and MDA‐MB‐231 hypoxia‐inducible factor (HIF) reporter cell lines were transduced and selected as previously described.^[^ ^33^ ^]^ For all cultures, passage number did not exceed 20.

Human dendritic cells (DCs) were differentiated from cryopreserved CD14+ monocytes over a period of 7 days as previously described.^[^ ^79^ ^]^ In brief, monocytes were seeded into a 6‐well plate in ImmunoCult‐ACF Dendritic Cell Medium (Stemcell Technologies), supplemented with recombinant human granulocyte‐macrophage colony‐stimulating factor (GM‐CSF) (50 ng/mL) (R&D Systems) and recombinant human interleukin‐4 (IL‐4) (50 ng mL^−1^) (R&D systems). For pericellular O_2_ studies, DCs were cultured in a 96‐well plate with 200 µL with 750,000 cells mL^−1^.

Primary human hepatocytes (HUCPG, Lonza) were cultured following the manufacturer's protocol. Briefly, hepatocytes were thawed in thawing medium (MCHT50, Lonza) and then seeded onto a 24‐well plate (BioCoat Collagen I, Corning) using plating medium (MP100 and MP250, Lonza). The seeding process involved gentle shaking every 15 min for 1 h (Seed 1), followed by replacement of the plating medium and another 4 h of incubation (Seed 2). Subsequently, the hepatocytes were then cultured (T = 0 h) using maintenance medium (CC‐3198, Lonza), in either normoxia or physioxia (6% O_2_), which was exchanged after 24 h. Finally, cells were harvested for RNA‐seq after 36 h of culture.

For O_2_‐controlled experiments, cells were incubated within a tri‐gas incubator (Heracell VIOS 160i, Thermo Fisher Scientific), which was kept closed throughout the duration of the experiment. Cells were not removed from O_2_‐controlled environments for passaging. Cultures that were taken out of the tri‐gas incubator for processing were immediately placed on ice and lysed.

### Cellular Hypoxia Kinetics

MCF7 cells were seeded onto 60 mm glass dishes (Cellvis) overnight and incubated with Hoechst 33 342 (NucBlue Live ReadyProbes Reagent, Invitrogen), 10 µm Image‐iT Red Hypoxia Reagent (Invitrogen), and 1 µm Celltracker Orange for 30 min at 37 °C. Cells were placed within a humidified incubator at 37 °C, 5% CO_2_, and 1% O_2_/99% N_2_ (O_2_ Module S, CO_2_ Module S, and Temp Module S, Zeiss) attached to a confocal microscope (LSM 880 with Airyscan, Zeiss). Images were taken every 30 min for 12 h. The average fluorescence per cell was calculated using Fiji. Briefly, cell area multiplied by background fluorescence was subtracted from the cell's integrated density. At least thirty cells were analyzed per image.

### Hypoxia‐Inducible Factor (HIF) Stabilization Kinetics

For the cell density experiments, MCF7 HIF reporter cells were used as previously described.^[^ ^33^ ^]^ Briefly, these cells contain two vectors: Vector 1 consists of a Cre gene modified by the addition of an O_2_‐dependent degradation domain, which is transcriptionally controlled by a HIF‐DNA binding sequence (HRE). Vector 2 consists of a red fluorescent protein gene (Dsred) with a stop codon flanked by tandem loxP sites, followed by a green fluorescent protein gene (GFP). MCF7 reporter cells were seeded into a 12‐well plate at different densities (7000 and 29000 cells cm^−2^) overnight. Cells were cultured at 1% O_2_ for 4 days, incubated with Hoechst 33 342, and confocal images were taken daily. The percentage of GFP+ among MCF7 reporter cells was determined using Fiji (Threshold and Analyze Particles). For culture vessel type experiments, MDA‐MB‐231 HIF reporter cells were seeded onto a 24‐well plate or T25 flask (30000 cells cm^−2^) and cultured at 1% O_2_ for 5 days. Flow cytometry (Cytoflex S, Beckman Coulter) of live singlets was used to determine the GFP‐positive fraction.

### Mitochondrial Inhibition

MCF7 were seeded overnight in a 24‐well plate and incubated at 5% O_2_ for 24 h. 10 µL of DPBS or sodium azide (Sigma–Aldrich) (final concentration = 5 mm) were added into cultures. For cytotoxicity studies, MCF7 were seeded overnight and incubated with ± 5 mm sodium azide for 6 h at 5% O_2_. Cells were incubated with 1:1000 live/dead dye (LIVE/DEAD Fixable Kit, Thermo Fisher Scientific) for 1 h. Flow cytometry (Attune NxT, Thermo Fisher Scientific) of live singlets was used to determine the cell viability.

### Cell Viability Time Course

MCF7 were seeded into a T25 flask (20000 cells cm^−2^) overnight and cultured at 18.6%, 5%, or 1% O_2_ for 6 days. Every 2 days, media was harvested, and trypan blue staining and a hemocytometer were used to determine live and dead detached cells. Attached cells were trypsinized (Trypsin‐EDTA, Gibco) and counted using the same method.

### Library Preparation with polyA Selection and Illumina Sequencing

RNA was extracted immediately from hepatocytes (NucleoSpin RNA, Macherey‐Nagel) and quantified using a Qubit 2.0 Fluorometer (Life Technologies). Cells were removed from O_2_‐controlled incubators, immediately placed on ice, and then lysed using Lysis Buffer RA1. RNA integrity was checked using Agilent TapeStation 4200 (Agilent Technologies). RNA sequencing libraries were prepared using the NEBNext Ultra II RNA Library Prep for Illumina per the manufacturer's protocol (New England Biolabs). Briefly, mRNAs were enriched with Oligod(T) beads. Enriched mRNAs were fragmented for 15 min at 94 °C. First‐strand and second‐strand cDNA were subsequently synthesized. cDNA fragments were end‐repaired and adenylated at 3′ ends, and universal adapters were ligated to cDNA fragments, followed by index addition and library enrichment by PCR with limited cycles. The sequencing libraries were validated on the Agilent TapeStation 4200 and quantified by using Qubit 2.0 Fluorometer as well as by quantitative PCR (KAPA Biosystems). The sequencing libraries were multiplexed and clustered onto a flowcell. After clustering, the flowcell was loaded onto the Illumina instrument (HiSeq 400 or equivalent) according to the manufacturer's instructions. The samples were sequenced using a 2 × 150 bp Paired End (PE) configuration. Image analysis and base calling were conducted by the HiSeq Control Software (HCS). Raw sequence data (.bcl files) generated from Illumina HiSeq were converted into FASTQ files and de‐multiplexed using Illumina bcl2fastq 2.20 software. One mismatch was allowed for index sequence identification. FASTQ files were trimmed with Trimmomatic^[^ ^80^ ^]^ and the quality was analyzed with FastQC. The human genome (GRCh38.p14) was annotated and reads were aligned using STAR.^[^ ^81^ ^]^ Gene counts were determined using FeatureCounts.^[^ ^82^ ^]^ Differential gene expression analysis was performed using DESeq2.^[^ ^83^ ^]^ Gene set enrichment analysis (GSEA)^[^ ^84^ ^]^ was performed using the clusterProfiler package in R.^[^ ^85^ ^]^

### Reaction‐Diffusion Model

The unsteady state diffusion Equation (1) with initial and boundary conditions ((2), (3), (4)) were used to describe O_2_ transfer between cell culture media and gas phase, where C is the concentration of O_2_, D is the diffusivity coefficient, and k_L_a is the mass transfer coefficient. x = 0 is the bottom of the well and x = L is the media height. A diffusivity coefficient of 0.09684 cm^2^ h^−1^ was used^[^ ^44^ ^]^ and experimental diffusion data were used to determine k_L_ values. An analytical solution was determined ((5), (6)). Michaelis–Menten kinetics were used for the reaction‐diffusion model^[^ ^43^ ^]^ (7), where V_max_ is the maximum O_2_ consumption rate and K_m_ is the O_2_ concentration at which the reaction rate is half of V_max_. Numerical values were determined using the MATLAB PDE solver (MathWorks).

(1) $$\frac{\mathrm{dC}}{\mathrm{dt}}=\;D\frac{\partial^{2}C}{\partial x^{2}}\;$$

(2) $$C\left(x,t\;=\;0\right)=\;C_{0}\;$$

(3) $$\frac{dC\left(x\;=\;0,\;t\right)}{dx}=\;0\;$$

(4) $$-D\frac{\mathrm{dC}\left(x\;=\;L,\;t\right)}{\mathrm{dx}}=\;k_{L}a\left(C-C_{\mathrm{gas}}\right)\;$$

(5) $$\Theta \;\;\left(\zeta ,\tau \right)=\;\sum_{m\;=\;1}^{\infty }\frac{2\sin\left(\lambda_{m}\right)e^{-{\lambda_{m}}^{2}\tau }\cos\left(\lambda_{m}\zeta \right)}{\cos\left(\lambda_{m}\right)\sin\left(\lambda_{m}\right)+\;\lambda_{m}}\;\;$$

(6) $$\Theta \;=\;\frac{C-\;C_{gas}}{C_{0}-C_{gas}}\;\zeta \;=\;\frac{x}{L}\;\tau \;=\;\frac{Dt}{L^{2}}\;$$

(7) $$\frac{\mathrm{dC}}{\mathrm{dt}}=\;D\frac{\partial^{2}C}{\partial x^{2}}-\frac{V_{\max}C}{K_{M}+C}\;$$

### Extracellular Metabolite Quantification

Media was removed from cell cultures and centrifuged at 250 g for 5 min. The resulting supernatant was stored at −20 °C and used for metabolite quantification. Live cells from cultures were determined using trypan blue staining and a hemocytometer. Glucose uptake and lactate secretion were quantified by an Agilent 1260 high‐performance liquid chromatography (HPLC) Infinity II System equipped with a BioRad Aminex HPX‐87H ion exchange column (300 mm × 7.8 mm) operated at 60 °C with a refractive index detector (RID) operated at 50 °C.^[^ ^86^, ^87^ ^]^ The mobile phase was 14 mm sulfuric acid with a flow rate of 0.6 mL min^−1^. The injection volume of each sample was 10 µL. Peak areas for each compound for concentrations ranging from 0.125 to 5 g L^−1^ were used to make calibration curves in OpenLab ChemStation (LTS 01.11) and then used to calculate metabolite quantifications. Glutamate and glutamine concentration was determined using the Glutamine/Glutamate‐Glo Assay (Promega). Metabolite concentration was normalized by the cell number at each time point.

### Reverse Transcription and Quantitative PCR (RT‐qPCR)

RT‐qPCR was performed as previously described.^[^ ^88^ ^]^ RNA was extracted from MCF7 cultures (NucleoSpin RNA, Macherey‐Nagel). Cells were removed from O_2_‐controlled incubators, immediately placed on ice, and then lysed using Lysis Buffer RA1. RNA quality was checked using a NanoDrop One spectrophotometer (Thermo Fisher Scientific). Reverse transcription was conducted using a High‐Capacity cDNA Reverse Transcription Kit (Applied Biosystems) on a MyCycler thermal cycler (Bio‐Rad). Gene expression was quantified using the following TaqMan Gene Expression Assays (Thermo Fisher Scientific) on an MX3005P QPCR System (Agilent Technologies): *VEGF‐A* (Hs00900055_m1), *CA9* (Hs00154208_m1), *LDHA* (Hs01378790_g1), *PDK1* (Hs01561847_m1), *NT5E* (Hs00159686_m1), *PRKAA2* (Hs00178903_m1), *BNIP3L* (Hs00188949_m1), *BNIP3* (Hs00969291_m1), *HIF1A* (Hs00153153_m1), *HIF2A* (Hs00909569_g1), *SLC2A1* (Hs00892681_m1), *SLC1A5* (Hs01056542_m1), *SLC7A11* (Hs00921938_m1), *NDUFA4L2* (Hs00220041_m1), *BNIP3* (Hs00969291 _m1), *BNIP3L* (Hs00188949_m1), *NT5E* (Hs00159686_m1), *CD274* (Hs00204257_m1), and *ACTB* (Hs01060665_g1).

### Bulk Proteomic Sample Preparation

Cells were removed from O_2_‐controlled incubators, immediately placed on ice, centrifuged at 4 °C, resuspended in Mass Spectrometry grade Water (Fisher Scientific, W6500), and then frozen at −80 °C. Cells were lysed by heating at 90 °C for 10 min,^[^ ^89^ ^]^ and protein concentrations for each lysate were measured using a Nanodrop (A205). Proteins were digested to peptides per the SCoPE2 protocol.^[^ ^90^, ^91^ ^]^ Briefly, 10 µg of protein per sample were digested in a solution containing 100 mm triethylammonium bicarbonate at pH 8.5 (TEAB) (Sigma Aldrich, T7408), benzonase nuclease (Millipore Sigma, Cat E1014) and Trypsin Gold (Promega, V5280). The protease was added at a 1:20 enzyme‐to‐substrate ratio and LC‐MS grade water was added to maintain its concentration at 20 ng µL^−1^. The reaction was carried out for 12 h at 37 °C.

Digested peptides were subsequently dried down in a SpeedVac vacuum evaporator and resuspended in 200 mm TEAB (pH 8.5). Samples were randomized and labeled using either d0, d4, or d8 of mTRAQ mass tags (SciEx, 4 440 015, 4 427 698, and 4 427 700) in a reaction that maintained 1/3rd organic phase and the manufacturer's suggested reagent to peptide ratio (1U for 100 µg of peptides). The labeling was carried out for 2 h at room temperature and the excess, unreacted label was quenched by adding hydroxylamine (Sigma–Aldrich, 467 804) to 0.2% v/v and leaving at room temperature for 1 h. Two samples from each label were randomly selected, and 50 ng were analyzed in data‐dependent acquisition (DDA) mode to evaluate labeling efficiency.

Samples from each label were combined in equal amounts to make a plexDIA^[^ ^60^ ^]^ set that was dried down and resuspended in 0.1% formic acid (Thermo Fisher, 85 178) in MS grade water to a final concentration of 1 µg µL^−1^. Samples within a plexDIA set were randomly paired; a few samples across labels were repeated across multiple sets.

### Proteomics Data Acquisition

The separation was performed at a constant flow rate of 200 nL min^−1^ using a Dionex UltiMate 3000 UHPLC, and 1 µL of sample was loaded onto a 25 cm × 75 µm IonOpticks Odyssey Series column (ODY3‐25075C18). The separation gradient was 4% buffer B (80% acetonitrile in 0.1% Formic Acid) for 11.5 min, a 30 s ramp up to 12%B followed by a 63 min linear gradient up to 32%B. Subsequently, buffer B was ramped up to 95% over 2 min and maintained as such for 3 additional min. Finally, buffer B was dropped to 4% in 0.1 min and maintained for 19.9 additional min.

The mass spectra were analyzed using a Thermo Scientific Q‐Exactive mass spectrometer from min 20 to 95 of the LC method. An electrospray voltage of 1700 V was applied at the liquid‐liquid junction of the analytical column and transfer line. The temperature of the ion transfer tube was 250 °C, and the S‐lens RF level was set to 30.

Bulk data was collected in Data Independent Acquisition (DIA) mode, the duty cycle consisted of a total of 3 MS1 scans and 30 MS2 scans. All MS1 scans were conducted at 140000 resolving power with a maximum injection time of 300 ms and a target AGC of 3e6 with a scan range covering 378–1290 m z^−1^. All MS2 scans were conducted at 35000 resolving power, a maximum inject time of 110 ms, AGC target of 3e6, and normalized collision energy of 27. MS2 scans had variable isolation widths: 10 MS2 scans of 17 m z^−1^ isolation width (isolation window) followed the first and second MS1 scans respectively, the third MS1 was followed by 5 windows of 33 m z^−1^, 2 windows of 40 m z^−1^, 1 window of 80 m z^−1^ and a final window of 120 m z^−1^.

### Proteomics Data Processing

DIA‐NN^[^ ^92^ ^]^ (version 1.8.1) was used to search the raw files from each run. A predicted spectral library was made using the Swissprot mouse FASTA database and in silico labeled to have mTRAQ as a fixed mod (+140.0949630177) on each trypsin digested peptide.

Peak height was used for quantification with a scan window of 1, mass accuracy of 10 ppm and MS1 accuracy of 5 ppm, MBR was enabled, and search outputs were filtered at 1% Q value. The following commands were employed by use of the additional commands dialogue: –fixed‐mod mTRAQ 140.0949630177, nK, –channels mTRAQ, 0, nK, 0:0; mTRAQ, 4, nK, 4.0070994:4.0070994; mTRAQ, 8, nK, 8.0141988132:8.0141988132*}*, –peak‐translation, –ms1‐isotope‐quant, ‐ms1‐base‐profile, ‐ms1‐subtract 2.

The report file containing filtered peptide level output was processed using R. First, the peptide level data was collapsed/summarized to a run x protein matrix using the diannmaxlfq function from the “diann” R package.^[^ ^92^ ^]^ Subsequently, the matrix was log_2_ transformed and the protein levels in each run were normalized for differential loading amounts by adding to each protein value, the median of the difference between the vector of protein levels for that run to the vector of median values across all runs. Relative protein levels were obtained by subtracting away the mean value across runs for each protein. In order to correct for biases specific to each mTRAQ label, kNN imputation (k = 3) was performed and ComBat^[^ ^93^ ^]^ was used with mTRAQ labels as batch covariates. Post batch correction two matrices were used for further analysis, one with imputed values and the other where the imputed values had been set back to NA. Differential protein expression analysis was performed using limma.^[^ ^94^ ^]^ Protein set enrichment analysis (PSEA)^[^ ^84^ ^]^ was performed using the clusterProfiler^[^ ^85^ ^]^ package in R.

### Statistical Analysis

All data were presented as the mean ± standard error of the mean (SEM). Biological replicate indicates a unique culture for a given condition. Statistical analyses were performed using Prism 9 software (GraphPad). The number of replicates and statistical tests used are outlined in the figure captions. Values represent the mean ± standard error of the mean. Significance levels are reported as ^*^ *p* < 0.05, ^**^ *p* < 0.01, ^***^ *p* < 0.001, ^****^ *p* < 0.0001.

## Conflict of Interest

The authors declare no conflict of interest.

## Author Contributions

Z.J.R., T.C., and S.A.B. conceived the presented idea and designed the experiments. Z.J.R. conducted the experiments unless otherwise stated. T.C. conducted the MCF7 transcription analysis. S.K. conducted the proteomics experiments and N.S. supported the analysis. K.B. and A.N. conducted the dendritic cell experiments. B.W. supported the development of the computational model. G.Z. conducted the glucose and lactate quantitation. C.T. and D.G. helped design the kinetic and pericellular measurement experiments and supported the transcriptional and translational analysis. Z.J.R. wrote the manuscript and generated the figures. All authors discussed the results, commented on, and proofread the manuscript. The principal investigator is S.A.B.

## Supporting information

Supporting Information

## Data Availability

The data that support the findings of this study are available from the corresponding author upon reasonable request.
