# Supplementary material for: Controlling Pericellular Oxygen Tension in Cell Culture Reveals Distinct Breast Cancer Responses to Low Oxygen Tensions
Source: Adv Sci (Weinh). 2024 Jun 14;11(30):2402557. doi: 10.1002/advs.202402557 (PMC11321643; doi:10.1002/advs.202402557)
Supplement: Supplementary file 1 — Supporting Information [file ADVS-11-2402557-s001.pdf]

ADVANCED SCIENCE
Open Access

Supporting Information

for *Adv. Sci.*, DOI 10.1002/advs.202402557

Controlling Pericellular Oxygen Tension in Cell Culture Reveals Distinct Breast Cancer Responses to Low Oxygen Tensions

*Zachary J. Rogers, Thibault Colombani, Saad Khan, Khushbu Bhatt, Alexandra Nukovic, Guanyu Zhou, Benjamin M. Woolston, Cormac T. Taylor, Daniele M. Gilkes, Nikolai Slavov and Sidi A. Bencherif**

Supplementary Information

Controlling Pericellular Oxygen Tension in Cell Culture Reveals Distinct Breast Cancer Responses to Low Oxygen Tensions

Zachary J. Rogers[1], Thibault Colombani[1], Saad Khan[2], Khushbu Bhatt[3], Alexandra Nukovic[1], Guanyu Zhou[1], Benjamin M. Woolston[1], Cormac T. Taylor[4], Daniele M. Gilkes[5,6,7,8], Nikolai Slavov[9,10], Sidi A. Bencherif[1,2,11,12,‡]

[1] Department of Chemical Engineering, Northeastern University, Boston, MA 02115, USA

[2] Department of Bioengineering, Northeastern University, Boston, MA 02115, USA

[3] Department of Pharmaceutical Sciences, Northeastern University, Boston, MA 02115, USA

[4] Conway Institute of Biomolecular and Biomedical Research and School of Medicine, University College Dublin, Belfield, Dublin, D04 V1W8, Ireland

[5] Department of Oncology, The Sidney Kimmel Comprehensive Cancer Center, The Johns Hopkins University School of Medicine, Baltimore, MD 21321, USA

[6] Cellular and Molecular Medicine Program, The Johns Hopkins University School of Medicine, Baltimore, MD 21321, USA

[7] Department of Chemical and Biomolecular Engineering, The Johns Hopkins University, Baltimore, MD 21218, USA

[8] Johns Hopkins Institute for NanoBioTechnology, The Johns Hopkins University, Baltimore, MD 21218, USA

[9] Departments of Bioengineering, Biology, Chemistry and Chemical Biology, Single Cell Center and Barnett Institute, Northeastern University, Boston, MA 02115 USA

[10] Parallel Squared Technology Institute, Watertown, MA 02135 USA

[11] Harvard John A. Paulson School of Engineering and Applied Sciences, Harvard University, Cambridge, MA 02138, USA

[12] Biomechanics and Bioengineering (BMBI), UTC CNRS UMR 7338, University of Technology of Compiègne, Sorbonne University, 60203 Compiègne, France

[‡] Corresponding author: s.bencherif@northeastern.edu

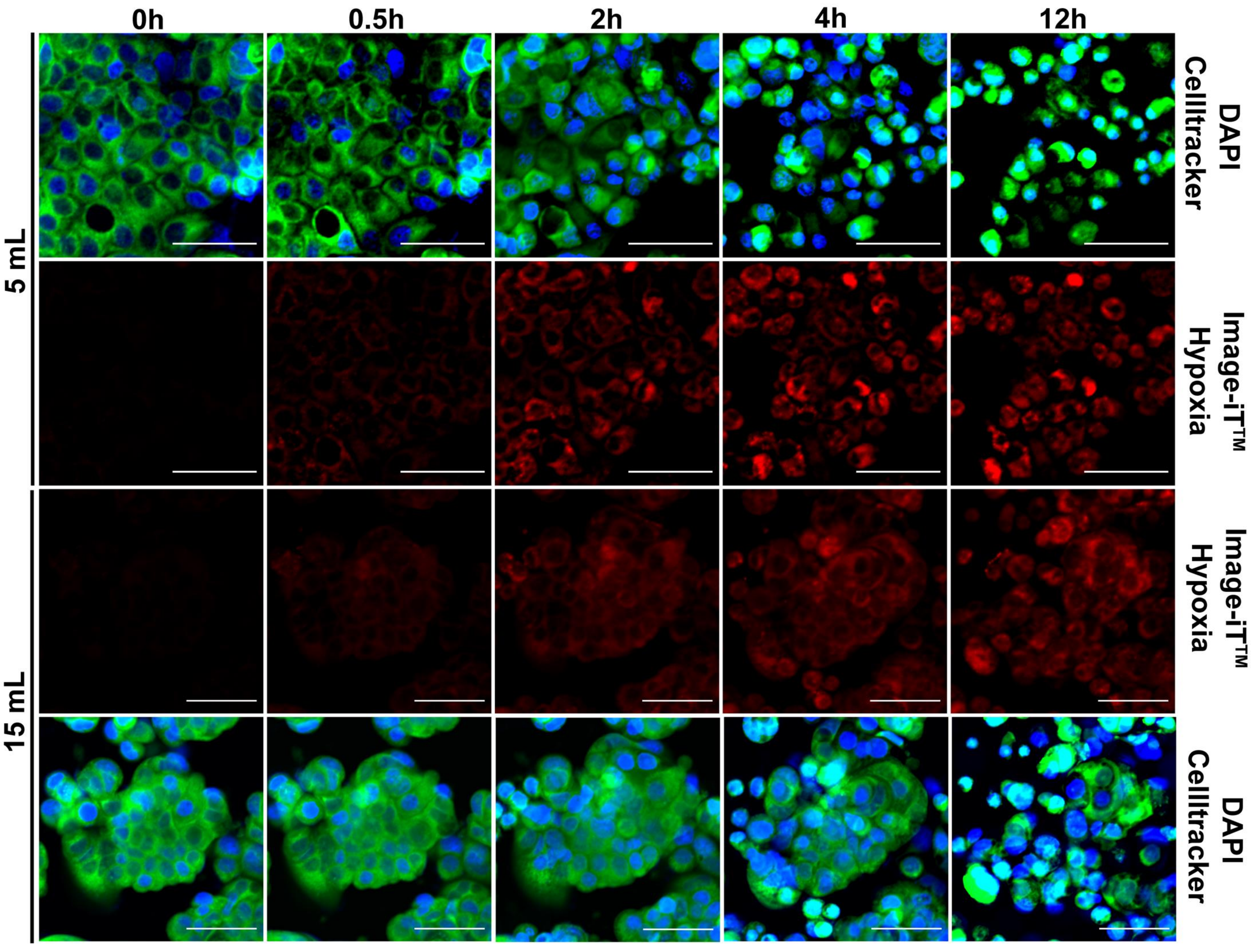

0h
0.5h
2h
4h
12h
5 mL
15 mL
DAPI
Celltracker
Image-iT™
Hypoxia
Image-iT™
Hypoxia
DAPI
Celltracker

Figure S1: Medium volume influences cellular hypoxia kinetics in 1% O_2 culture. Representative confocal images of MCF7 cultures in 60mm dishes with 5 mL or 15 mL of medium placed inside a 1% O_2 chamber for 12h. Confocal images were taken every 30min. Red = Image-iT^{TM} Hypoxia as an indicator of cellular hypoxia. Blue = nuclei stained with DAPI. Green = cytoplasm stained with Celltracker. N = 3 biological replicates per condition.

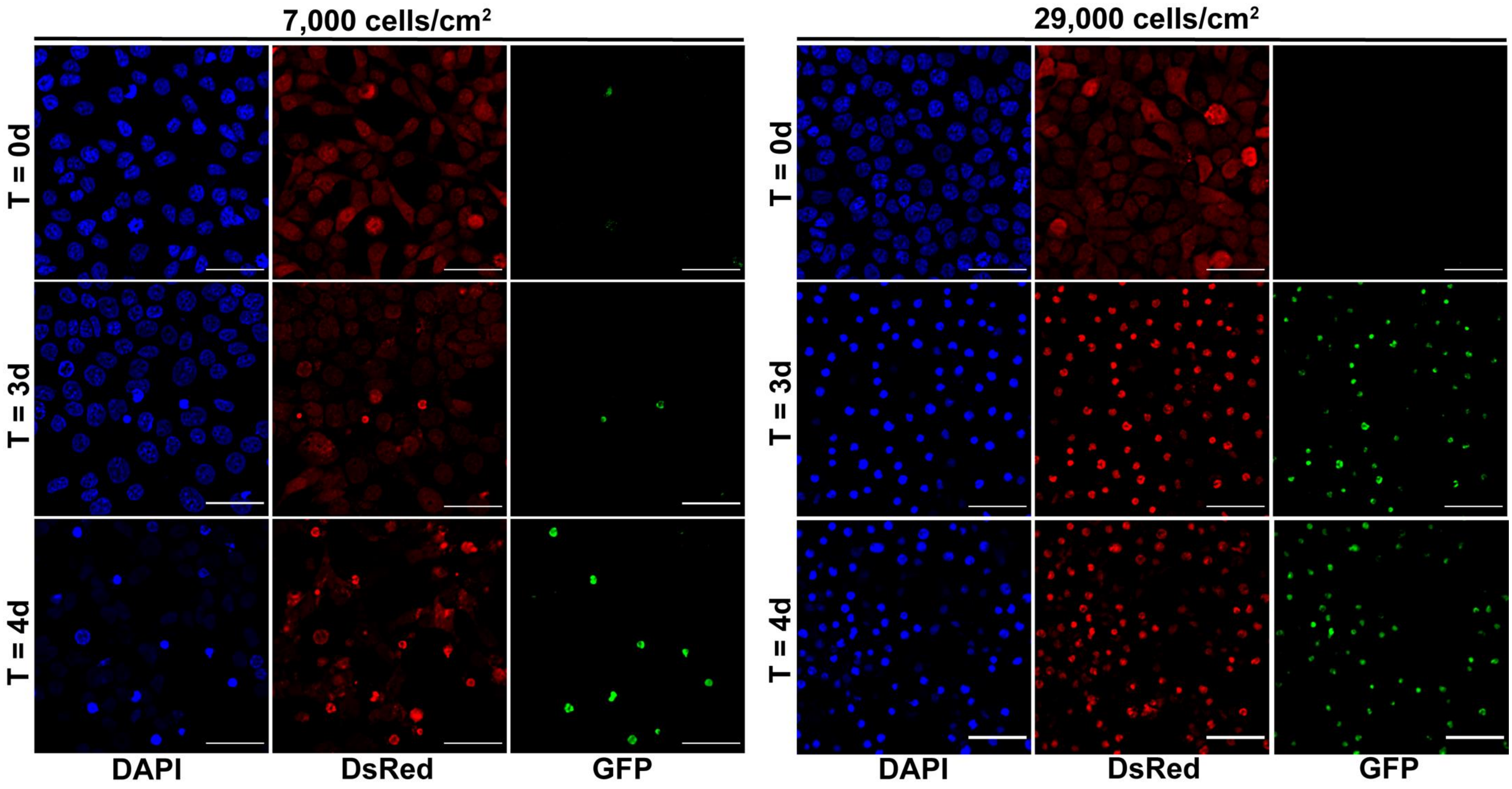

7,000 cells/cm2
29,000 cells/cm2
T = 0d
T = 3d
T = 4d
T = 0d
T = 3d
T = 4d
DAPI
DsRed
GFP
DAPI
DsRed
GFP

Figure S2: Cell density influences HIF stabilization kinetics in 1% O_2 culture. Representative confocal images of MCF7 HIF reporter cells cultured at different densities (7,000 and 29,000 cells/cm^2) in a 1% O_2 incubator for 4 days. Images were taken at 0, 3 and 4 days. Different replicates were used for each time point to prevent reoxygenation. Blue = nuclei stained with DAPI. Red = Dsred (HIF-). Green = GFP (HIF+). N = 4 biological replicates per condition.

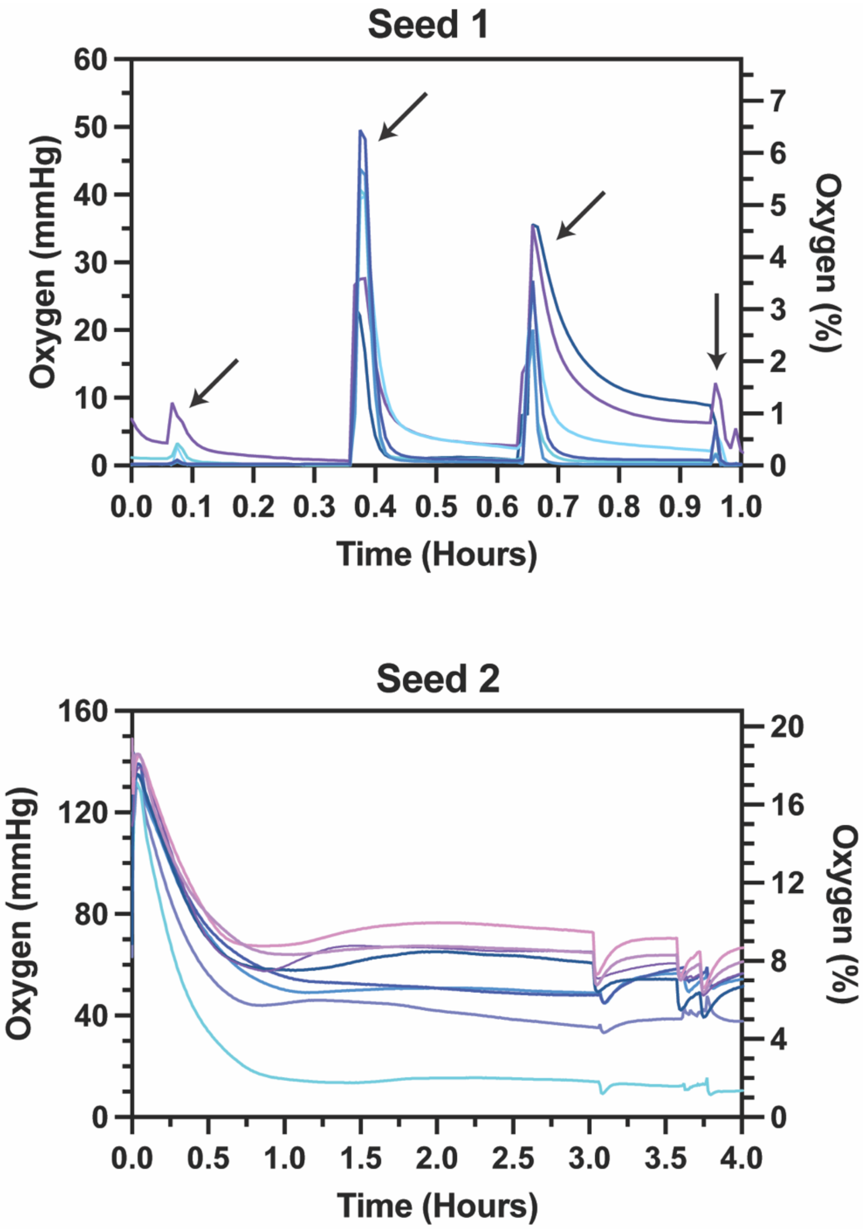

Figure S3: Primary human hepatocytes experience anoxia during seeding in normoxia. Top: O_2 kinetics during the 1st seeding step of hepatocytes into a Collagen I 24-well plate for 1 hour. Arrows indicate when plates were shaken to promote seeding. **Bottom:** O_2 kinetics during the 2nd seeding step of hepatocytes for 4 hours. N = 6 – 8 biological replicates.

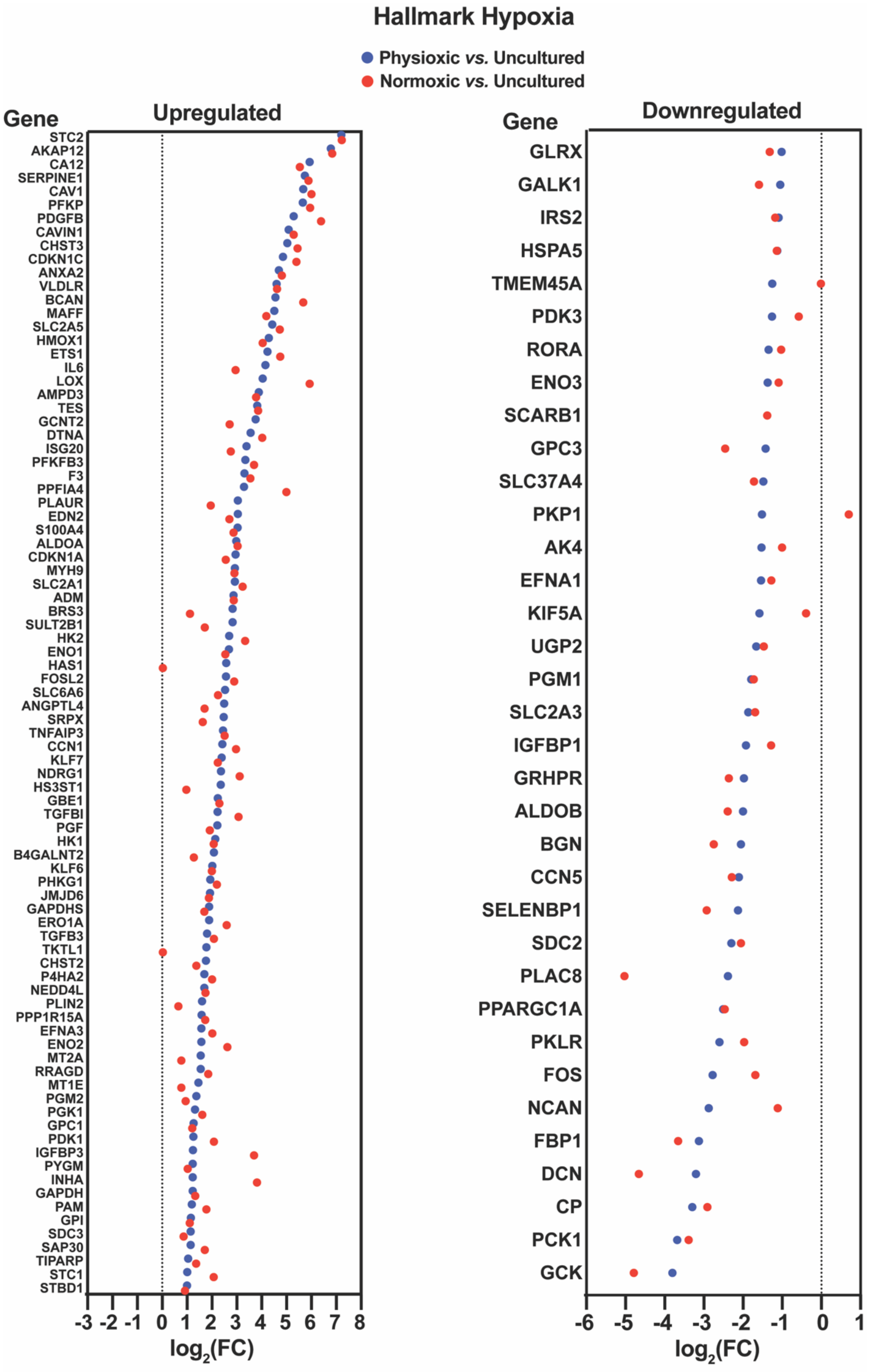

Hallmark Hypoxia
Physioxic vs. Uncultured
Normoxic vs. Uncultured
Gene
Upregulated
STC2
AKAP12
CA12
SERPINE1
CAV1
PFKP
PDGFB
CAVIN1
CHST3
CDKN1C
ANXA2
VLDLR
BCAN
MAFF
SLC2A5
HMOX1
ETS1
IL6
LOX
AMPD3
TES
GCNT2
DTNA
ISG20
PFKFB3
F3
PPFIA4
PLAUR
EDN2
S100A4
ALDOA
CDKN1A
MYH9
SLC2A1
ADM
BRS3
SULT2B1
HK2
ENO1
HAS1
FOSL2
SLC6A6
ANGPTL4
SRPX
TNFAIP3
CCN1
KLF7
NDRG1
HS3ST1
GBE1
TGFBI
PGF
HK1
B4GALNT2
KLF6
PHKG1
JMJD6
GAPDHS
ERO1A
TGFB3
TKTL1
CHST2
P4HA2
NEDD4L
PLIN2
PPP1R15A
EFNA3
ENO2
MT2A
RRAGD
MT1E
PGM2
PGK1
GPC1
PDK1
IGFBP3
PYGM
INHA
GAPDH
PAM
GPI
SDC3
SAP30
TIPARP
STC1
STBD1
-3 -2 -1 0 1 2 3 4 5 6 7 8
log2(FC)
Gene
Downregulated
GLRX
GALK1
IRS2
HSPA5
TMEM45A
PDK3
RORA
ENO3
SCARB1
GPC3
SLC37A4
PKP1
AK4
EFNA1
KIF5A
UGP2
PGM1
SLC2A3
IGFBP1
GRHPR
ALDOB
BGN
CCN5
SELENBP1
SDC2
PLAC8
PPARGC1A
PKLR
FOS
NCAN
FBP1
DCN
CP
PCK1
GCK
-6 -5 -4 -3 -2 -1 0 1
log2(FC)

Figure S4: Primary human hepatocytes cultured in normoxia and physioxia exhibit a similar hypoxic transcriptomic response. Upregulated (left) and downregulated (right) genes in the Hallmark Hypoxia gene set for physioxic *vs.* uncultured (blue) and normoxic *vs.* uncultured (red) cultures. Genes with $|\log_2(FC)| < 1$ for physioxic *vs.* uncultured are not shown.

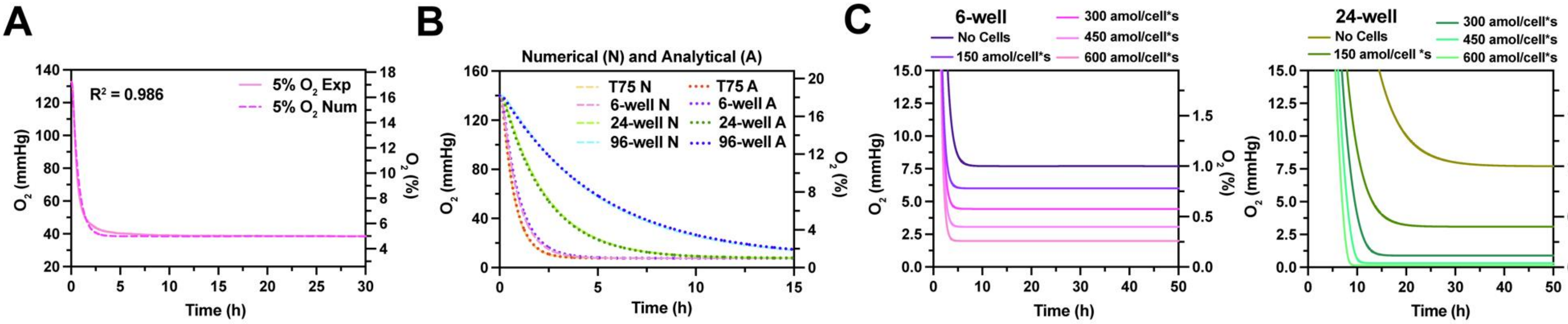

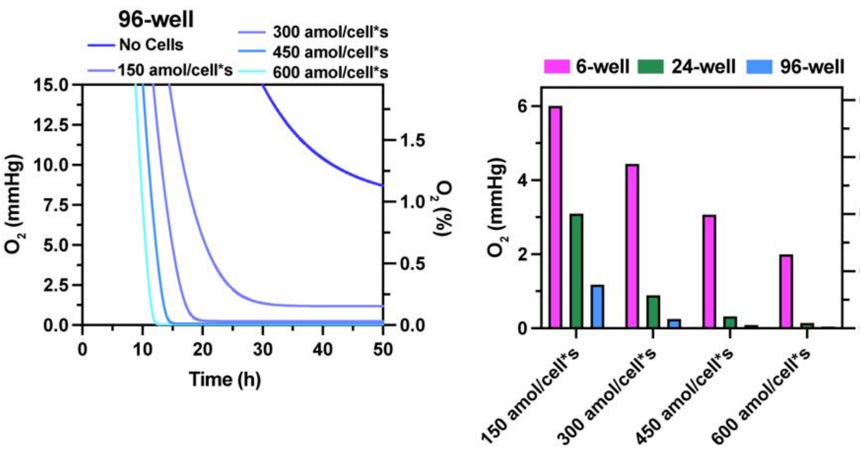

D

Vessel	Volume (mL)	Surface Area to Height (cm)
6-well	1	92.6
	2	46.3
	3	30.9
24-well	0.5	8.5
	0.75	5.7
	1	4.2
96-well	0.1	1.4
	0.15	0.9
	0.2	0.7

Figure S5: Developing a reaction-diffusion model to predict pericellular O_2 tension in cell culture. (A) Experimental (solid) and numerical (dashed) O_2 kinetics of media (EMEM + 10% FBS + 1% P/S) placed in a 5% O_2 incubator. **(B)** Numerical (N) (dashed) and analytical (A) (dotted) O_2 kinetics for media in different culture vessel types placed in a 1% O_2 incubator. **(C)** Reaction-diffusion model predictions of cells (20,000 cells/cm^2) with different O_2 consumption rates (V_{max}) in a 6-well, 24-well, or 96-well plate in a 1% O_2 incubator. **(D)** Surface area to height ratios for 6-well, 24-well, or 96-well plates given commonly used volumes for each culture vessel.

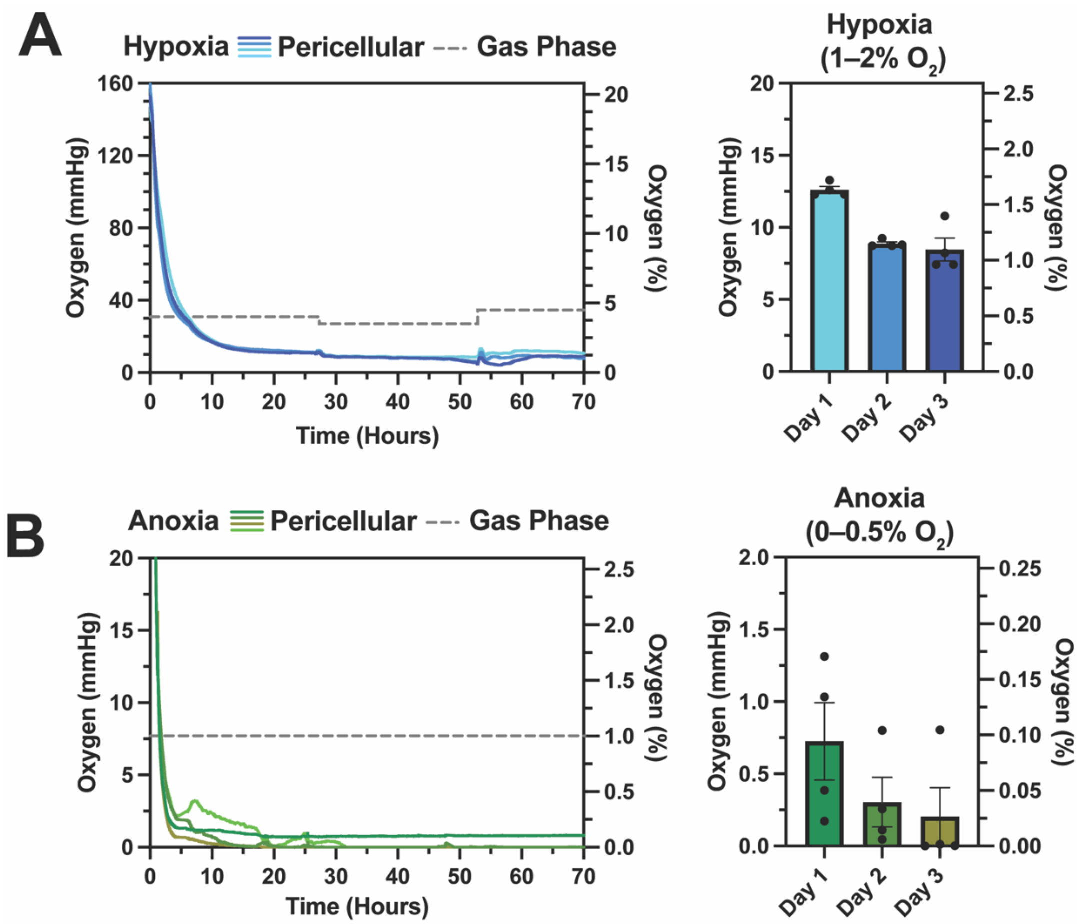

Figure S6: Controlling pericellular hypoxia and anoxia in MCF7 cultures. (A–B) Pericellular (solid) and gas phase (dashed) O_2 kinetics (left) and average O_2 tensions (right) for MCF7 cultures targeting pericellular hypoxia (1–2% O_2) **(A)** and anoxia (0–0.5% O_2) **(B)**. Gas phase O_2 tension was manually changed to maintain pericellular hypoxia in **(A)**. MCF7 was cultured in 6-well plates with 70,000 cells per well. N = 4 biological replicates per condition.

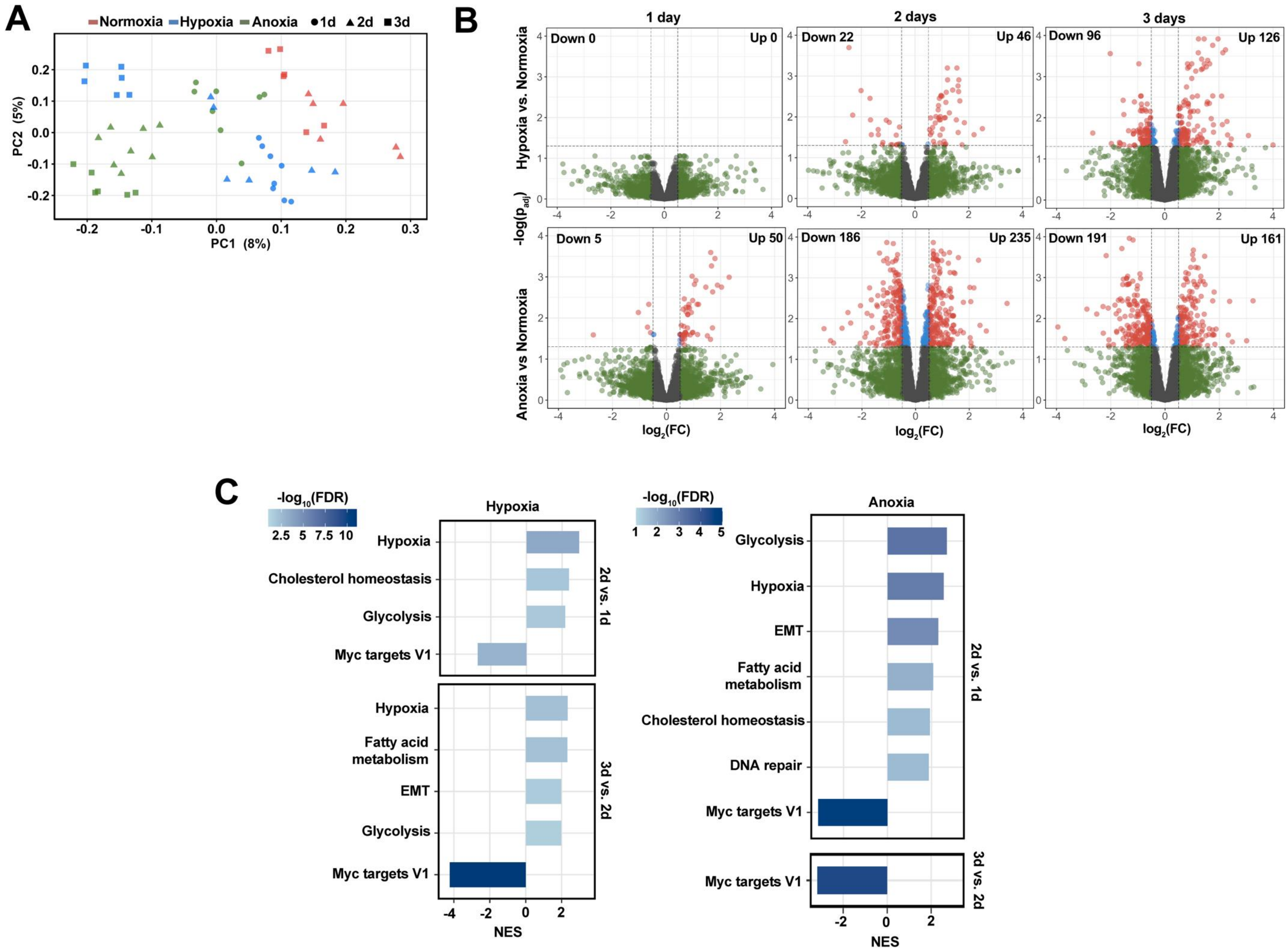
A
Normoxia Hypoxia Anoxia 1d 2d 3d
PC2 (5%)
PC1 (8%)
B
1 day
2 days
3 days
Hypoxia vs. Normoxia
Anoxia vs Normoxia
-log(p_adj)
log2(FC)
Down 0
Up 0
Down 22
Up 46
Down 96
Up 126
Down 5
Up 50
Down 186
Up 235
Down 191
Up 161
C
-log10(FDR)
Hypoxia
Hypoxia
Cholesterol homeostasis
Glycolysis
Myc targets V1
2d vs. 1d
Hypoxia
Fatty acid metabolism
EMT
Glycolysis
Myc targets V1
3d vs. 2d
NES
Anoxia
Glycolysis
Hypoxia
EMT
Fatty acid metabolism
Cholesterol homeostasis
DNA repair
Myc targets V1
2d vs. 1d
Myc targets V1
3d vs. 2d
NES

Figure S7: Proteomic characterization of the temporal differences between pericellular hypoxic and anoxic responses in 4T1. (A) Principal component analysis (PCA) of protein abundances for 4T1 cells cultured in different O_2 tensions for 1, 2 or 3 days of culture. **(B)** Volcano plots indicating signficant downregulated or upregulated proteins ($p_{adj} < 0.05$ and $|log_2FC| \geq 0.5$) for Hypoxia *vs.* Normoxia (top) or Anoxia *vs.* Normoxia (bottom) for 1 day, 2 days, or 3 days of culture. **(C)** PSEA using the Hallmark database, comparing different days in hypoxia (left) or anoxia (right). N = 6–8 replicates per condition. NES = normalized enrichment score. N = 3 biological replicates and N = 2–3 technical replicates per condition.
